# ALDH5A1-deficient iPSC-derived excitatory and inhibitory neurons display cell type specific alterations

**DOI:** 10.1016/j.nbd.2023.106386

**Published:** 2023-12-16

**Authors:** Wardiya Afshar-Saber, Nicole A. Teaney, Kellen D. Winden, Hellen Jumo, Xutong Shi, Gabrielle McGinty, Jed Hubbs, Cidi Chen, Itay Tokatly Latzer, Federico Gasparoli, Darius Ebrahimi-Fakhari, Elizabeth D. Buttermore, Jean-Baptiste Roullet, Phillip L. Pearl, Mustafa Sahin

**Affiliations:** a Rosamund Stone Zander Translational Neuroscience Center, Boston Children’s Hospital, Harvard Medical School, Boston, MA, USA; b FM Kirby Neurobiology Center, Department of Neurology, Boston Children’s Hospital, Harvard Medical School, Boston, MA, USA; c Washington State University, Department of Pharmacotherapy, Spokane, WA, USA; d Department of Cell Biology, Harvard Medical School, Boston, MA, USA; e Human Neuron Core, Rosamund Stone Zander Translational Neuroscience Center, Boston Children’s Hospital, Boston, MA, USA; f Faculty of Medicine, Tel-Aviv University, Tel-Aviv, Israel

**Keywords:** Epilepsy, Autism spectrum disorder, Stem cell derived neurons, GABA metabolism, Mitochondrion

## Abstract

Succinic semialdehyde dehydrogenase deficiency (SSADHD) is a neurometabolic disorder caused by ALDH5A1 mutations presenting with autism and epilepsy. SSADHD leads to impaired GABA metabolism and results in accumulation of GABA and γ-hydroxybutyrate (GHB), which alter neurotransmission and are thought to lead to neurobehavioral symptoms. However, why increased inhibitory neurotransmitters lead to seizures remains unclear. We used induced pluripotent stem cells from SSADHD patients (one female and two male) and differentiated them into GABAergic and glutamatergic neurons. SSADHD iGABA neurons show altered GABA metabolism and concomitant changes in expression of genes associated with inhibitory neurotransmission. In contrast, glutamatergic neurons display increased spontaneous activity and upregulation of mitochondrial genes. CRISPR correction of the pathogenic variants or SSADHD mRNA expression rescue various metabolic and functional abnormalities in human neurons. Our findings uncover a previously unknown role for SSADHD in excitatory human neurons and provide unique insights into the cellular and molecular basis of SSADHD and potential therapeutic interventions.

## 1. Introduction

Succinic semialdehyde dehydrogenase deficiency (SSADHD) is a rare metabolic disorder caused by loss-of-function mutations of the *ALDH5A1* gene (Pearl et al., 2015). Clinically, it manifests as early onset encephalopathy associated with intellectual disability, ataxia, and ASD (Pearl et al., 2011). In addition, over half of the patients develop intractable seizures and have a high risk of sudden unexpected death in epilepsy (SUDEP) (Pearl et al., 2011). SSADH is a mitochondrial enzyme that, in tandem with γ-aminobutyric acid (GABA) transaminase, catabolizes GABA to succinic acid. In SSADHD, GABA accumulates and is converted to γ-hydroxybutyric acid (GHB), leading to a pathognomonic increase of both metabolites in the blood, urine, and tissues (Wamelink et al., 2011; Jansen et al., 2016). GABA is the major inhibitory neurotransmitter in the central nervous system (CNS), and GHB is a strong CNS depressant, although its precise mechanism of action remain unclear (Gibson et al., 2005a). Studies of individuals with SSADHD and animal models of the disorder have demonstrated significant down-regulation of multiple types of GABA receptors, and these changes are thought to be compensatory due to high levels of GABA signaling (Pearl et al., 2009; Errington et al., 2011). Interestingly, the production of GABA and GHB has been shown to decrease with age (DiBacco et al., 2019), which has led to the hypothesis that dwindling inhibitory neurotransmitters in the face of down-regulated receptors results in paradoxically reduced inhibition and hyperexcitable neuronal circuits (Pearl et al., 2009; Tokatly Latzer et al., 2023). Vigabatrin is an approved seizure medication that irreversibly inhibits GABA transaminase. Treatment with vigabatrin leads to the expected rise in both the free and total GABA levels in CSF, with a concomitant decrease in GHB levels (Casarano et al., 2012). Although there are no controlled trials of vigabatrin in SSADHD, case studies have reported mixed results with use of vigabatrin in patients with SSADHD in terms of seizure control (Gropman, 2003). Therefore, it is likely that there are non-GABAergic mechanisms contributing to epilepsy in SSADHD.

The ability to reprogram somatic cells into pluripotent stem cells coupled with advances in techniques to differentiate these stem cells into specific cell types has been highly advantageous for the study of neurodevelopmental disorders (Winden et al., 2019; Rosa et al., 2020; Mossink et al., 2022). The ability to use specific transcription factors to induce development of populations of neurons that are highly enriched for specific sub-types allows for molecular and biochemical dissection of pathological processes *in vitro* that would be far more difficult *in vivo* (Yang et al., 2017; Zhang et al., 2013). To better understand the impact of SSADHD on the development of the disease phenotypes in GABAergic and glutamatergic neurons, we generated both neuronal subtypes from patient-derived induced pluripotent stem cells (iPSCs). Interestingly, we observed that loss of SSADH causes neuron subtype-specific metabolic and gene expression changes. Our data suggest that loss of ALDH5A1 activity in excitatory neurons may also play an important role in the development of aberrant neuronal networks in SSADHD.

## 2. STAR methods

## 3. Method details

### 3.1. Study design

This study aimed to generate an *in vitro* model of SSADHD using hiPSC-derived neurons to investigate SSADHD-related phenotypes. Human Subjects ethics committee Boston Children’s Hospital Institutional Review Board (IRB) approved the protocol (IRB-P00016119) to study hiPSC lines at the Boston Children’s Hospital (Boston, MA, USA). Three patients, three parental controls, and a CRISPR-corrected line were used for this study. hiPSC lines were reprogrammed from the donor fibroblasts, and the CRISPR-Cas9-edited *ALDH5A1*^corr/corr^ line was generated by the Human Genome Editing Core at UConn Health. In this study, Family 001 hiPSCs were derived from a patient with SSADHD due to homozygote Fam001-*ALDH5A1*^−/−^, c.1226G > A; p.Gly409Asp and the sex-matched parental control heterozygote Fam001-*ALDH5A1*^+/−^. An additional hiPSC line (Fam005-*ALDH5A1*^corr/corr^) was generated from Fam001-*ALDH5A1*^−/−^ using the CRISPR-Cas9 system. Family 002 hiPSCs were derived from a patient with SSADHD due to homozygote Fam002-*ALDH5A1*^−/−^, c.612G > A, p.Trp204Ter and the sex-matched parental control heterozygote Fam002-*ALDH5A1*^+/−^. Family 003 hiPSCs were derived from a patient with SSADHD due to compound heterozygous Fam003-*ALDH5A1*^−/−^, c.612G > A, p.Trp204Ter and the sex-matched parental control heterozygote Fam003-*ALDH5A1*^+/−^. The number of replications varied and is indicated in each figure legend (*n* ≥ 3). The cell lines used for experimentation were not blinded; however, analyses were conducted using automated software such as for MEA, calcium imaging, neurite outgrowth. Data is available from the corresponding author upon reasonable request. All iPSC lines generated in this study are available with a material transfer agreement.

### 3.2. Statistical analyses

Statistical analyses were performed with GraphPad Prism version 9.4.0. We performed parametric or nonparametric tests between groups: two-way analysis of variance followed by Tukey’s multiple comparisons test or Kruskal–Wallis test followed by Dunn’s multiple comparisons tests, and *p*-values were adjusted for multiple comparisons. Unpaired *t*-tests (parametric and nonparametric) with one- and two-tailed p-values were performed in cases where comparisons were independent. *P*-values are presented as follows: *p < 0.05, **p < 0.01, ***p < 0.001, and ****p < 0.0001. Figure legends include the details of the statistical methods used to analyze each dataset.

### 3.3. Human iPSC lines

The hiPSC lines used in this study were transduced with pLV-TetO-hNGN2-P2A-mCherry-T2A-Puro or pLV-TetO-hDLX2-P2A-hASCL1-T2A-Puro resulting in the generation of 10 iPSC lines in total. These vectors were a gift from Aleksandar Bajic (Baylor College of Medicine) (Fig. S1). The iPSC lines were confirmed to have normal karyotypes by G-banded karyotyping, and colonies were analyzed for the expression of pluripotency markers NANOG, TRA1–60, OCT4, and SSEA4 (Fig. S2). These lines were used for differentiation and phenotypic characterization. The hiPSC colonies were maintained in mTeSR^™^ Plus cGMP supplemented with 1× mTeSR^™^ Plus Supplement (Stem Cell Technologies # 100–0276) on Vitronectin (rhVTN-N A14700) coated plates and passaged every 4–5 days, and the media were changed every other day. Cells were tested routinely to be mycoplasma negative. hiPSCs were utilized for 10 passages out of thaw from confirmed karyotype before a new vial was thawed and a max of 32 passages per line was used.

### 3.4. Generation of CRISPR-corrected ALDH5A1^corr/corr^: SSADH gRNAs and templates

Sequence PAM Location Strand

*ALDH5A1*–1 GGTGCCACCGTTGTGACAG**A** TGG chr6:24528030–−24528052 +

*ALDH5A1*–2 TCGTTTTCCA**T**CTGTCACAA CGG chr6:24528037–−24528059−

Ultramer ssODN templates from IDT - ssODN templates:

SSADH_G409-A (bottom strand—use with either *ALDH5A1*–1 or − 2) gtctcttcatgagtgcacagcatgtcctgggtgacattgcacagcagggtaggctcaaagaaattttttccaagttggtgtcgttttccA**C**CtgtcacaacTgtggcaccttta-gaaacggcatcattcacctgtttctccacctgtgtaatgaggaagaaaaaaagctttccacatg.

SSADH_G409-B (top strand—use with *ALDH5A1*–1) cattctaaaa-gattgtatcatgtggaaagctttttttcttcctcattacacaggtggagaaacaggtgaatgatgccgtttctaaaggtgccacAgttgtgacaG**G**Tggaaaacgacaccaacttggaaaaaatttctttgagcctaccctgctgtgcaatgtcacccaggacatgctg. Colour key: silent mutation; **A ≥ G correction**.

### 3.5. Neuronal differentiation

To generate iGABA and iNs, we used protocols previously published by Südhof and colleagues using induced NGN2 expression with minor changes and Wernig and colleagues using induced DLX2 and ASCL1 with minor changes. At DIV0, hiPSCs were dissociated into single cells with Accutase (catalog #AT 104–500; Innovative Cell Technology) and seeded onto Geltrex-coated plates at a density of 100,000/cm^2^ (catalog #A1413301; Thermo Fisher Scientific). hiPSCs were then infected at DIV1 with concentrated rtTA-, hNGN2-expressing lentiviruses or rtTA-, and hDLX2-hASCL1-expressing lentiviruses in the presence of polybrene (8 μg/ml, catalog #TR-1003-G; Sigma-Aldrich, FUW-M2rtTA addgene #20342) overnight. At DIV3, hiPSCs were fed with supplemented mTeSRPlus and expanded for cryopreservation. In parallel, we performed a kill curve to determine the optimal puromycin concentration to eliminate non-transduced cells. We also tested the successful transduction by adding doxycycline to the infected cells for 24 h (2 μg/ml, catalog #324385–1GM; Millipore) followed by the optimized puromycin concentration (1 μg/ml, catalog #ant-pr-1; Invitrogen) for up to 48 h. To generate iGABA neurons, the hDLX2-hASCL1 transduced hiPSCs were dissociated into single cells and seeded onto Geltrex-coated plates. At DIV1, hDLX2-hASCL1 expression was induced using doxycycline and selected with puromycin, similarly to the efficiency test until DIV6. iGABA neurons were then dissociated with Accutase supplemented with DNaseI and replated onto Poly-D-Lysine (PDL 0.5 mg/ml; Sigma Aldrich P6407) and laminin (5 μg/ml; Life Technologies 23,017–015) coated plates with or without hiPSC-derived astrocytes (Astro.4 U; Ncardia) at densities specific for each phenotypic endpoint, as described below. iGABA neurons were fed every other day B27 medium supplemented with doxycycline (2 μg/ml) (DIV7–14), followed by the addition of Ara- C (4uM) for five days (DIV14–20). iGABA neurons were then fed every other day with B27 medium supplemented with growth factors BDNF (10 ng/ml, catalog #450–02; Peprotech), GDNF (10 ng/ml, catalog #450–10; Peprotech), and laminin (1 μg/l, catalog #23017–015; Thermo Fisher Scientific) and cultured for various durations until they were assayed, as indicated below. To generate iNs, the hNGN2 transduced hiPSCs were dissociated into single cells using Accutase and seeded onto Geltrex-coated plates at a density of 100,000/cm^2^. The next day, hNGN2 expression was induced using doxycycline and selected with puromycin, similar to the efficiency test. Growth factors BDNF (10 ng/ml, catalog #450–02; Peprotech), NT3 (10 ng/ml, catalog #450–03; Peprotech), and laminin (0.2 mg/l, catalog #23017–015; Thermo Fisher Scientific) were added in N2 medium for the first two days. Cells were then fed with BDNF (10 ng/ml), NT3 (10 ng/ml), laminin (0.2 mg/l), doxycycline (2 μg/ml), and Ara-C (4uM, catalog #C1768; Sigma- Aldrich) in B27 media and fed every other day until dissociation at DIV6. Cells were then dissociated with papain (catalog #LK003178; Worthington) and DNaseI (catalog #LK003172; Worthington) and replated on Poly-D-Lysine (PDL 0.5 mg/ml; catalog P6407; Sigma Aldrich) and laminin (5 μg/ml; catalog #23017–015; Life Technologies) coated plates with or without hiPSC-derived astrocytes (Astro.4 U; Ncardia) at densities specific for each phenotypic endpoint, as described below. Cultures were maintained for various durations until they were assayed, as indicated below.

### 3.6. Mycoplasma testing

All cellular cultures were routinely tested for mycoplasma by PCR. Media supernatants (with no antibiotics) were collected, centrifuged, and resuspended in a saline buffer. Ten microliters of each sample were used for a PCR with the followings sets of primers: Myco280_CReM (5′- ACACCATGGGAGYTGGTAAT-3′); Myco279_CReM (5′-CTTCWTCGACTTYCAGACCCAAGGCAT-3′) from the Center for Regenerative Medicine: CReM - Boston University and MGSO-5 (5′-TGCACCATCTGTCACTCYGTTAACCTC-3′) and GPO-3 (5′- GGGAGCAAACAGGATTAGA-TACCCT-3′). Only negative samples were used in the study.

### 3.7. Immunocytochemistry

15-mm round coverslips (catalog #229172; CELLTREAT) were placed at the bottom of 24-well plates (Costar^®^ 24-well Clear TC-treated Multiple Well Plates, catalog #3524, Corning) and coated with poly-D-lysine (100 μg/ml, catalog #P6407; Sigma-Aldrich) overnight at room temperature. Wells were washed three times with DNase/RNase-free distilled water (catalog #10977015; Invitrogen) and allowed to dry completely. Wells were coated with natural mouse laminin (10 μg/ml, catalog #23017–015; Life Technologies) overnight at 37 °C.

### 3.8. GABA or TBR1, MAP2, DAPI in iGABA or iN cultures

200,000 iGABA or iNs were dissociated at DIV6 and replated on PDL/laminin-coated glass coverslips with 15% astrocytes (catalog #M0605; NCardia) in each well. Cells were maintained in culture for 50 days before fixing with 8% PFA added to media at 50:50 volume at room temperature. Cells were washed thrice with DPBS (Dulbecco’s Phosphate Buffered Saline, catalog *#*D8537*)*. Cells were blocked with 5% normal goat serum (catalog # AB_2336983, Jackson Immuno Research), 2% BSA (catalog #BSA-10, Rockland), and 0.1% TritonX-100 (catalog #AC215682500; Troy Biologicals) for one hour. Primary antibodies rabbit anti-GABA for iGABA neurons (1:500, catalog #A2052; Sigma- Aldrich), or rabbit anti-TBR1 for iNs (1:500, catalog # ab31940; Abcam) and chicken anti-MAP2 (1:2000, catalog #ab5392; Abcam) were diluted in blocking buffer and incubated overnight at 4 °C. Cells were washed three times before incubation of fluorophore-conjugated secondary antibodies for one hour at room temperature. Secondary antibodies, Alexa Fluor 647^™^ goat anti-rabbit IgG (H + L) (1:100, catalog #A21244; Invitrogen), and Alexa Fluor^™^ 488 goat anti-chicken IgG (H + L) (1:100, catalog #A11039; Invitrogen), were diluted in blocking buffer. Cells were washed three times with DPBS and counterstained with Hoechst (4 μg/ml, catalog #H3569; Invitrogen) in DNase/RNase- free distilled water for 5 min at room temperature. Cells were washed twice with DNase/RNase-free distilled water prior to mounting on glass slides with ProLong^™^ Diamond Antifade Mountant (catalog #P36965; Invitrogen). We imaged the stained coverslips with a Yokogawa CSU-W1 spinning disk confocal installed on a Nikon Ti-E microscope. We used the Hamamatsu Orca-Fusion BT camera with a Nikon Plan Apo 20 × 0.75NA DIC M N2 objective. The laser used were as follows: 405 nm laser with a dichroic mirror Semrock Di01-T405/488/568/647 and emission filter Chroma ET455/50 m; 488 nm laser with a dichroic mirror Semrock Di01-T405/488/568/647 and emission filter Chroma ET525/ 50 m; 561 nm laser with a dichroic mirror Semrock Di01-T405/488/ 568/647 and emission filter Chroma ET605/52 m; 640 nm laser with a dichroic mirror Semrock Di01-T405/488/568/647 and emission filter Chroma ET705/72 m. We used the Nikon NIS Elements software for acquisition, and maximum projections of the images were created from the z-stacked images. Acquired images were handled using Fiji software (Schindelin et al., 2012).

### 3.9. Western blot analyses

The protein content of cultured iPSC-derived neurons was extracted using RIPA buffer with 1:100 PMSF (ChemCruz, 200 mM), phosphatase inhibitor (Selleckchem, 100 mM), and proteinase inhibitor (ChemCruz). Undissolved materials were removed using benchtop microcentrifuge (4 °C, 14,000 rpm, 20 min). Sample protein contents were measured by Pierce^™^ BCA Protein Assay Kit (catalog #23225; Thermo Scientific). Equal amounts of protein were loaded and resolved by polyacrylamide gel electrophoresis (PAGE) using Criterion TGX 4–20% Precast Gels (Biorad) and transferred onto PVDF membrane using iBlot gel transfer system (Invitrogen). Membranes were dried overnight and rehydrated with 100% Methanol, MilliQ Water, and TBS before blocking for one hour at room temperature in Intercept (TBS) blocking buffer (LI-COR). Membranes were then incubated for one hour at room temperature with primary antibodies (rabbit anti-SSADH, mouse anti-β-actin), washed by TBS-T, incubated for one hour at room temperature with IRDye 800CW anti-rabbit and 680RD anti-mouse secondary antibodies (LI-COR), and imaged using Odyssey imaging system (LI-COR). Images were processed and quantified using Image Studio acquisition software (LI-COR). Proteins of interest (*i.e*., SSADH) were normalized by β-actin internal control, then compared between proband, CRISPR-corrected, and experimental (parent) control. This experiment was repeated three times using three independently differentiated batches of neurons.

### 3.10. GHB and GABA quantification

Dissociated iGABA neurons were replated on PDL/laminin-coated 12-well plates at a density of 100,000/cm^2^. Supernatant were collected for GABA and GHB quantification using GABA (LDN, Nord-horn, Germany) and GHB enzyme-linked immunosorbent assay (ELISA) kits (BUHLMANN Diagnostics Corp, Amherst, NH) according to the vendor’s protocol.

### 3.11. mRNA transfection

Dissociated iGABA neurons were replated on PDL/laminin-coated 12-well plates at a density of 100,000/cm^2^ and differentiated for 35 days as described in “Neuronal differentiation”, (Fig. S1B). At DIV35, iGABA neurons were transfected with 1 μg mRNA coding for full-length human *ALDH5A1* (*hALDH5A1* mRNA, catalog #ENST00000357578.8; TriLink, 1 mg scale, 5-Methoxyuridine, 1 mM Sodium Citrate, pH 6.4), using Lipofectamine MessengerMAX mRNA Transfection (catalog #LMRNA001; Invitrogen) according to the vendor’s protocol (*n* = 3 independent transfection). Control eGFP was used following the same conditions (mRNA CleanCap^®^ EGFP mRNA Media, catalog # L-7601; TriLink, 1 mg scale 5-Methoxyuridine, 1 mM Sodium Citrate, pH 6.4). Supernatant and protein lysates were collected 72 h post transfection as described above.

### 3.12. Calcium imaging

Ninety-six well plates were coated with Poly-D-lysine hydrobromide 0.5 mg/ml (catalog #P6407; Sigma-Aldrich) at room temperature overnight, washed with DNase/RNase-free distilled water, dried overnight and laminin (5 μg/ml; catalog #23017–015; Life Technologies) overnight at 37 °C, 5% CO_2_. iNs were dissociated at DIV6 as above and replated with hiPSC-derived astrocytes at density of 50,000 iNs/cm^2^ and 3750 astrocytes/cm^2^ in media supplemented with Y-27632 (catalog #10005583; Cayman) and laminin (10 μg/ml). Cultures of iNs and hiPSC-derived astrocytes were transduced with lentiviral particles pLV- hSyn-jRCaMP1b at DIV 21, and recordings were performed at DIV77. Viral particles were prepared by the Viral Core at Boston Children’s Hospital. This experiment was repeated three times using three independently differentiated batches of neurons. The samples were imaged with a Nikon 20× NA 0.75 air objective using a Nikon Ti2E inverted microscope equipped with a pco.edge 4.2 sCMOS Camera with a 2 × 2 or 4 × 4 binning. The change in fluorescence from the GECI jRCaMP1b was recorded at 10 Hz under continuous green illumination at 550 nm through a filter set (excitation filter 565/24 nm, dichroic mirror 562 nm, longpass emission filter >570 nm). Analysis was performed using FluoroSNNAP (Patel et al., 2015).

### 3.13. Multi-electrode array (MEA)

Forty-eight well CytoView MEA plates (M768-tMEA-48B; Axion Biosystems) were coated with Poly-D-lysine hydrobromide 0.5 mg/ml (catalog #P6407; Sigma-Aldrich) at room temperature overnight, washed with DNase/RNase-free distilled water, dried overnight and then coated laminin (5 μg/ml; catalog #23017–015; Life Technologies) overnight at 37 °C, 5% CO_2_. iNs were dissociated at DIV6 as above, and 1.10 (Gibson et al., 2005a)/cm^2^ iNs and 15.10 (Wamelink et al., 2011)/cm^2^ hiPSC-derived astrocytes were resuspended in 20 μl of media supplemented with Y-27632 (catalog #10005583; Cayman) and laminin (10 μg/ml) and were manually spotted in each well. We differentiated *ALDH5A1*^−/−^, *ALDH5A1*^+/−^ and *ALDH5A1*^corr/corr^ lines in parallel. This experiment was repeated three times using three independently differentiated batches of neurons. For each replicate, 16 to 24 wells were dedicated to each genotype, with 16 electrodes per well. Spontaneous network activity was recorded with a Maestro-1 MEA System (Axion Biosystems) every other day for 50 days. To ensure the robustness of the data, we discarded any well with <25% active electrodes. Data were sampled at 12.5 kHz, digitized, and analyzed using Axion Integrated Studio software with a 200 Hz high-pass and 3000 Hz low-pass filter with an adaptive spike detection threshold set at six times the SD for each electrode with 1 s binning. The burst detector was set to detect network bursts, with a maximum of 100 ms interspike interval, a minimum of 50 spikes, and a minimum of 35% participating electrodes. The window size for synchrony parameters is set at 20 ms. After recording, the data were analyzed using the Neurostatistics compiler (Axion Biosystems).

### 3.14. Metabolomics

Samples were processed at the Beth Israel Deaconess Medical Center (BIDMC) Mass Spectrometry (Proteomics/Metabolomics) Core using a previously described protocol (Yuan et al., 2012). This experiment was repeated three times using three independently differentiated batches of neurons.

### 3.15. RNA Sequencing and data analysis

Total RNA was extracted from iGABA and iNs at the indicated time points using RNeasy Plus Kit (Qiagen, catalog #74034). Messenger RNA was purified from total RNA using poly-T oligo-attached magnetic beads. After fragmentation, the first strand cDNA was synthesized using random hexamer primers, followed by the second strand cDNA synthesis using either dUTP for directional library or dTTP for non-directional library. For the non-directional library, it was ready after end repair, A-tailing, adapter ligation, size selection, amplification, and purification. For the directional library, it was ready after end repair, A-tailing, adapter ligation, size selection, USER enzyme digestion, amplification, and purification. The library was checked with Qubit and real-time PCR for quantification and bioanalyzer for size distribution detection. Quantified libraries will be pooled and sequenced on Illumina platforms, according to effective library concentration and data amount. Raw data (raw reads) of fastq format were first processed through in-house perl scripts. In this step, clean data (clean reads) were obtained by removing reads containing adapter, reads containing ploy-N, and low-quality reads from raw data. At the same time, Q20, Q30, and GC content of the clean data were calculated. All the downstream analyses were based on clean data with high quality. Reference genome and gene model annotation files were downloaded from the genome website directly. The reference genome index was built using Hisat2 v2.0.5 and paired- end reads aligned to the reference genome using Hisat2 v2.0.5. Quantification of gene expression levels was performed using featureCounts v1.5.0-p3. Weighted Gene Co-expression Analysis was performed in R (4.0.2) using the WGCNA package. Gene count data was normalized using counts per million and log_2_ transformed. Genes with a median expression level > 2 were used for network analysis. For differential expression analysis, raw count data were analyzed using EdgeR. Gene ontology analysis was performed using DAVID (https://david.ncifcrf.gov/summary.jsp). Gene Set Enrichment Analysis was performed using the GSEA software (https://www.gsea-msigdb.org/gsea/index.jsp). ASD risk genes were obtained from SFARI (https://gene.sfari.org/), and only genes with a score of 1 were used for further analysis. This experiment was repeated three times using three independently differentiated batches of neurons.

### 3.16. Seahorse assay

iNeurons were seeded in PDL/laminin coated XF 96-well plates (Seahorse/Agilent) (20 wells/genotype) at 50,000 iNs/cm^2^ in 100 μl growth medium. The Seahorse XF Cell Mito stress test kit was used according to manufacturer’s instructions (Agilent) using Oligomycin (2 μM), FCCP (1 μM), and Rotenone/Antimycin A (0.5 μM) to determine basal respiration level, proton leak, maximal, spare, and non-mitochondrial respiratory capacity.

## 4. Results

### 4.1. Differentiation of human iPSCs derived from patients with SSADHD into GABAergic neurons

**Fig. 1.**
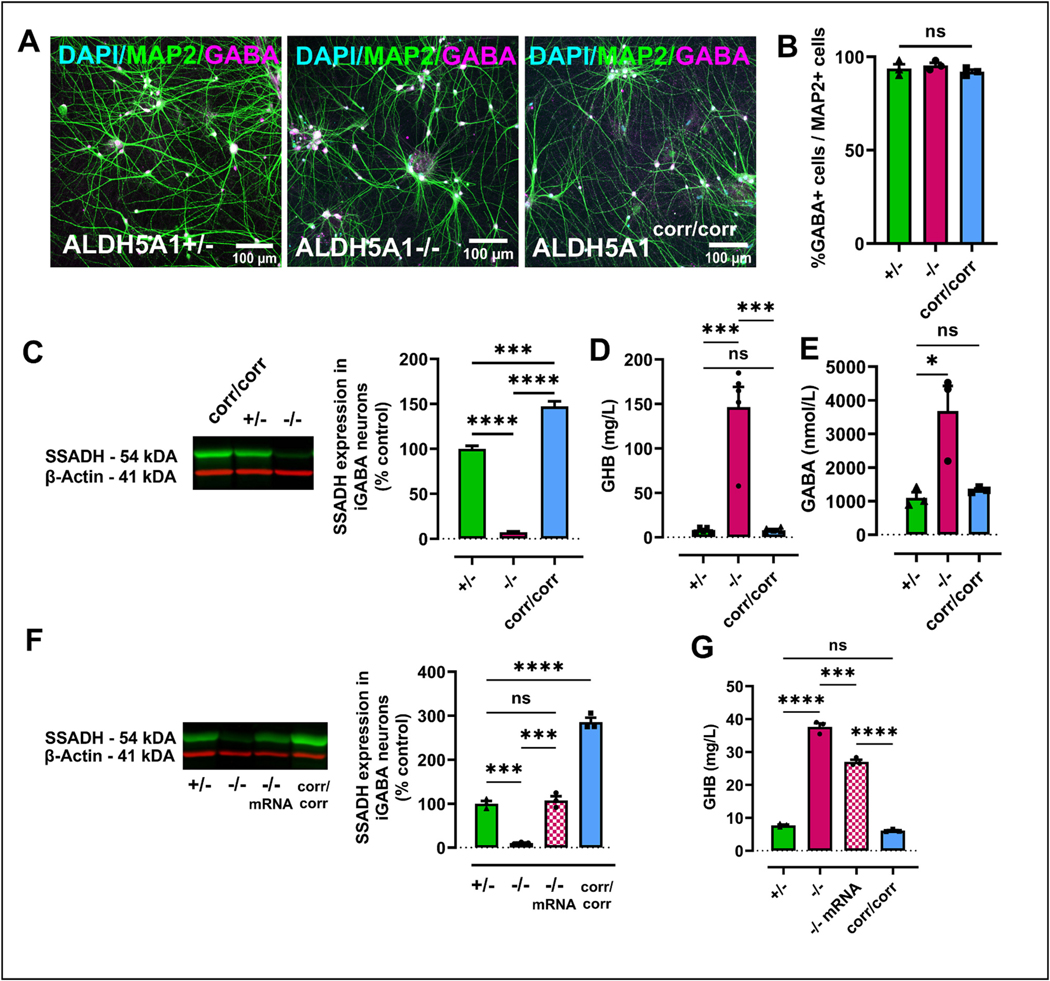
Differentiation of human iPSCs with SSADHD into GABAergic neurons. (A) Representative images of hiPSC-derived iGABA neurons from control (*ALDH5A1*^+/−^), patient (*ALDH5A1*^−/−^) and CRISPR corrected (*ALDH5A1*^corr/corr^), parental at DIV 55 in co-culture with hiPSC-derived astrocytes labelled with DAPI (cyan), MAP2 (green), GABA (magenta). Scale bar 100 μm. (B) Quantification of GABA- positive neurons over MAP2 positive neurons showing no significant difference between all three genotypes used in this study (*n* = 3). (C) Western blot of SSADH expression from iGABA neurons from all three genotypes at DIV 35. Quantification of SSADH levels from three separate differentiations displayed as percentage of parental control (*ALDH5A1*^+/−^) (mean ± s.e.m. values; n = 3; ****p < 0.0001; ***p < 0.001; F (2, 6) = 0.4781; one-way ANOVA with Tukey’s multiple comparisons test). (D) ELISA quantification of GHB in iGABA neurons at DIV 35 showing significant increase in *ALDH5A1*^−/−^ (*n* = 5 independent differentiations; two-way ANOVA; F (2, 8) = 39.68; ***p < 0.001, ns = non-significant). (E) ELISA quantification of GABA in iGABA neurons at DIV 35 showing significant increase in *ALDH5A1*^−/−^ (n = 3 independent differentiations; *p < 0.05; ns = non-significant; two-way ANOVA with Tukey’s multiple comparisons test; F (2, 4) = 8.675). (F) Western blot of SSADH expression from iGABA neurons of all three genotypes and SSADH-deficient 72 h after mRNA treatment (−/− treated). Quantification of SSADH levels normalized to β-actin and displayed as percentage of parental control (*ALDH5A1*^+/−^) (mean ± s.e.m. values; n = 3 separate transfection; ****p < 0.0001; ***p < 0.001; ns = non-significant; ANOVA with Tukey’s multiple comparisons test). (G) ELISA quantification of GHB in iGABA neurons at DIV 35 and after 72 h treatment with mRNA (−/− treated) reveals significant increase in *ALDH5A1*^−/−^ neurons (n = 3; ***p < 0.001, ns = non-significant) and decrease following mRNA treatment (mean ± s.e.m. values; n = 3 separate transfections; ****p < 0.0001; ***p < 0.001; ns = non-significant; two-way ANOVA with Tukey’s multiple comparisons test; F (3, 6) = 507.3).

We obtained skin fibroblasts from three unrelated patients with SSADHD and sex-matched parental controls. Parental controls were heterozygous for loss of function variants in *ALDH5A1*, while patients carried biallelic loss-of function variants: two were homozygous and one was compound heterozygous (Table 1). We reprogrammed primary cells from all six individuals hiPSCs using RNA viruses. An isogenic control line was generated by correcting both alleles of one proband’s hiPSCs using CRISPR-Cas9 gene editing (Fig. S1 A). We verified successful reprogramming by characterizing the expression of pluripotency markers (Fig. S2) and transduced the hiPSCs with lentiviral vectors that encode hDLX2 and hASCL1 (pLV-TetO-hDLX2-P2A-hASCL1-T2A-Puro) under the tetracycline promoter, as well as the puromycin resistance gene for selection to directly differentiate hiPSCs into iGABA neurons (Yang et al., 2017) (Fig. S1B). Seven hiPSC lines were karyotyped after transduction, and no abnormalities were detected (Fig. S2). hiPSCs were grown under feeder-free conditions, and on day 1 of differentiation, doxycycline was added to induce differentiation, followed by puromycin selection (Fig. S1B). We then cultured these differentiating cells in the presence of human iPSC-derived control astrocytes for 50 days to facilitate neuronal maturation and synapse formation. iGABA neurons expressed MAP2, as well as the neurotransmitter GABA (Fig. 1A and Fig. S3A, S3D). We observed the expression of these markers in all three genotypes, demonstrating that hiPSCs can differentiate into GABAergic neurons regardless of the allelic dosage of *ALDH5A1* under these conditions (Fig. 1B and Fig. S3A, S3D).

We next examined the expression of SSADH enzyme levels in iGABA neurons. Western blot analysis revealed a nearly complete absence of SSADH protein expression in *ALDH5A1*^−/−^ iGABA neurons compared to those derived from their parental control *ALDH5A1*^+/−^ (Fig. 1C and Fig. S3B, S3E). The CRISPR corrected *ALDH5A1*^corr/corr^ GABAergic neurons, which have biallelic expression of *ALDH5A1*, displayed restoration of expression of the SSADH protein compared to the *ALDH5A1*^−/−^ at higher levels than parental *ALDH5A1*^+/−^ lines. We used these *ALDH5A1*^corr/corr^ cells as an additional control as we phenotyped neurons with different levels of SSADH expression.

### 4.2. SSADHD results in increased GABA and GHB levels, which is rescued by mRNA transfection

In the absence of SSADH, transamination of GABA to succinic semialdehyde is followed by its reduction to GHB, leading to significant accumulation of GHB and GABA (Wamelink et al., 2011; Jansen et al., 2016). Therefore, we tested the effect of the SSADHD on GABA and GHB levels in iGABA neurons by ELISA. We found that hiPSC-derived GABAergic neurons produce GABA and GHB at detectable levels and that *ALDH5A1*^−/−^ iGABA neurons exhibited significantly higher levels of GABA and GHB (Fig. 1D, E, Fig. S3C and S3F) compared to their parental control. These findings confirm that SSADHD disrupts the GABA degradation pathway resulting in an accumulation of GABA and GHB and show that the iGABA neurons differentiated from patients recapitulate the biochemical phenotypes seen *in vivo* in patients with SSADHD.

Having established a robust human *in vitro* model of SSADHD, we tested the effectiveness of mRNA-based therapeutics to replace the deficient enzyme. We transfected iGABA neurons with mRNA coding for full-length human *ALDH5A1* (*hALDH5A1* mRNA). We confirmed the successful transfection using an mRNA-eGFP control and quantifying the GFP-positive neurons 72 h post-transfection (50% efficiency). We then tested whether transfection of *hALDH5A1* mRNA rescued SSADH protein expression and its metabolic activity. Western blot analysis revealed a rescue of the expression of the SSADH protein levels comparable to parental control iGABA neurons (Fig. 1F and Fig. S3D). This rescue was associated with a significant reduction of GHB 72 h post transfection (Fig. 1G and Fig. S3E). These data together with the CRISPR correction experiments further support our hypothesis of a direct link between genetic and metabolic dysfunction in the GABAergic neurons of patients with SSADHD.

### 4.3. Differentiation of hiPSCs derived from patients with SSADHD into glutamatergic neurons

While most studies have examined the effect of SSADHD on inhibitory neurons, ALDH5A1 is also highly expressed in excitatory cortical neurons based on single cell RNAseq data (Bakken et al., 2021). Thus, we sought to investigate the functional and molecular (gene expression) attributes of SSADH-deficient glutamatergic neurons. We used a transcription factor-based differentiation protocol that uses the induced expression of NGN2 to directly differentiate hiPSCs into glutamatergic neurons or iNeurons (iNs) (Zhang et al., 2013) (Fig. S1C). Seven hiPSC lines were transduced with lentiviral vectors that encode human NGN2 (hNGN2) under the tetracycline promoter, as well as the puromycin resistance gene for selection. hiPSCs were karyotyped after transduction, and no abnormalities were detected (Fig. S2). hiPSCs were grown under feeder-free conditions, and on day 1 of differentiation, doxycycline was added to induce differentiation, followed by puromycin selection.

**Fig. 2.**
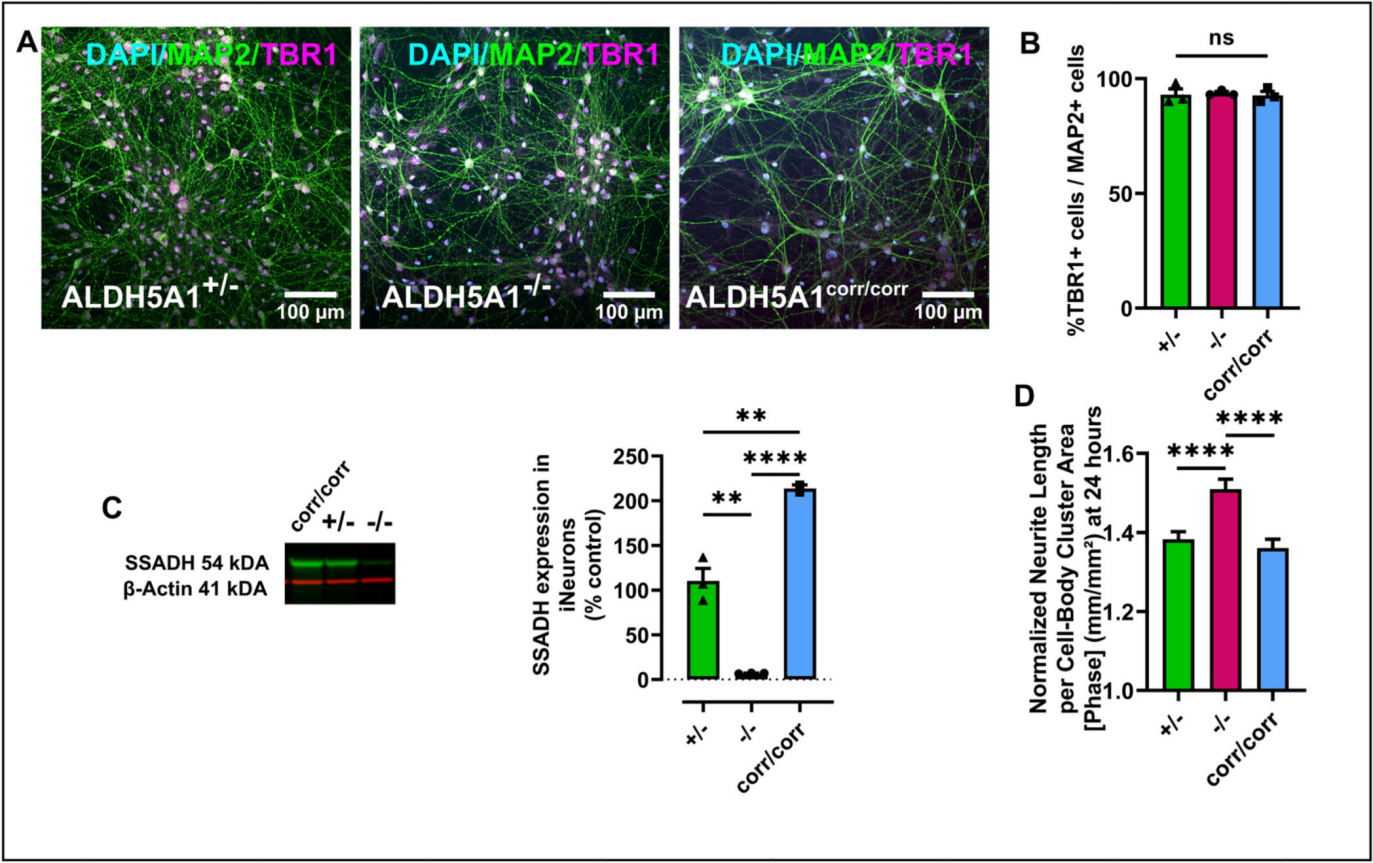
Differentiation and characterization of the SSADHD glutamatergic neurons. (A) Representative images of hiPSC-derived iNs parental control (*ALDH5A1*^+/−^), patient (*ALDH5A1*^− /−^) and CRISPR corrected (*ALDH5A1*^corr/corr^) at DIV 55 in co- culture with hiPSC-derived astrocytes labelled with DAPI (cyan), MAP2 (green) and TBR1 (magenta). Scale bar 100 μm. (B) Quantification of TBR1-positive neurons over MAP2-positive neurons showed no significant difference between all three genotypes used in this study (mean ± s.e.m. values; n = 3; ns = non-significant). (C) Western blot of SSADH expression from iNs of all three genotypes at DIV 30. Quantification of SSADH levels from three separate differentiations normalized to β-actin and displayed as a percentage of parental control (*ALDH5A1*^+/−^) (mean ± s.e.m. values; *n* = 2 for corr/corr and n = 3 for (+/−) and (−/−); ****p < 0.0001; **p < 0.01; F (2, 5) = 2.200; one-way ANOVA with Tukey’s multiple comparisons test). (D) Normalized iNs neurite length per cell-body cluster area [phase] (mm/mm^2^) at 24 h of all three genotypes demonstrates significantly longer neurites in *ALDH5A1*^−/−^ compared to controls (mean ± s.e.m. values; n = 3; ****p < 0.0001; ns = non-significant; one-way ANOVA with Tukey’s multiple comparisons test). (For interpretation of the references to colour in this figure legend, the reader is referred to the web version of this article.)

We then cultured these differentiating cells in the presence of human astrocytes for 50 days to facilitate neuronal maturation and synapse formation. These cells demonstrated expression of the mature neuronal marker MAP2 and the glutamatergic cortical marker TBR1 (Fig. 2A and Fig. S4A). We observed the expression of these markers in all three genotypes, demonstrating that hiPSCs can differentiate into cortical excitatory neurons regardless of the allelic dosage of *ALDH5A1* (Fig. 2B and Fig. S4A, S4I). Similar to iGABA neurons, biallelic loss of function mutations in *ALDH5A1* led to a near total loss of protein, while correction using gene editing (*ALDH5A1*^corr/corr^) led to expression above parental *ALDH5A1*^+/−^ lines (Fig. 2C). Additionally, we assessed the neurite outgrowth potential of iNs. The cells were replated at DIV6 and allowed to grow for 24h to assay neurite length. At 24h, the *ALDH5A1*^−/−^ neurites were significantly longer than *ALDH5A1^corr/corr^* and parental control *ALDH5A1*^+/−^ neurites (Fig. 2D and Fig. S4C).

### 4.4. SSADHD is linked with altered activity of the developing glutamatergic neuronal network

**Fig. 3.**
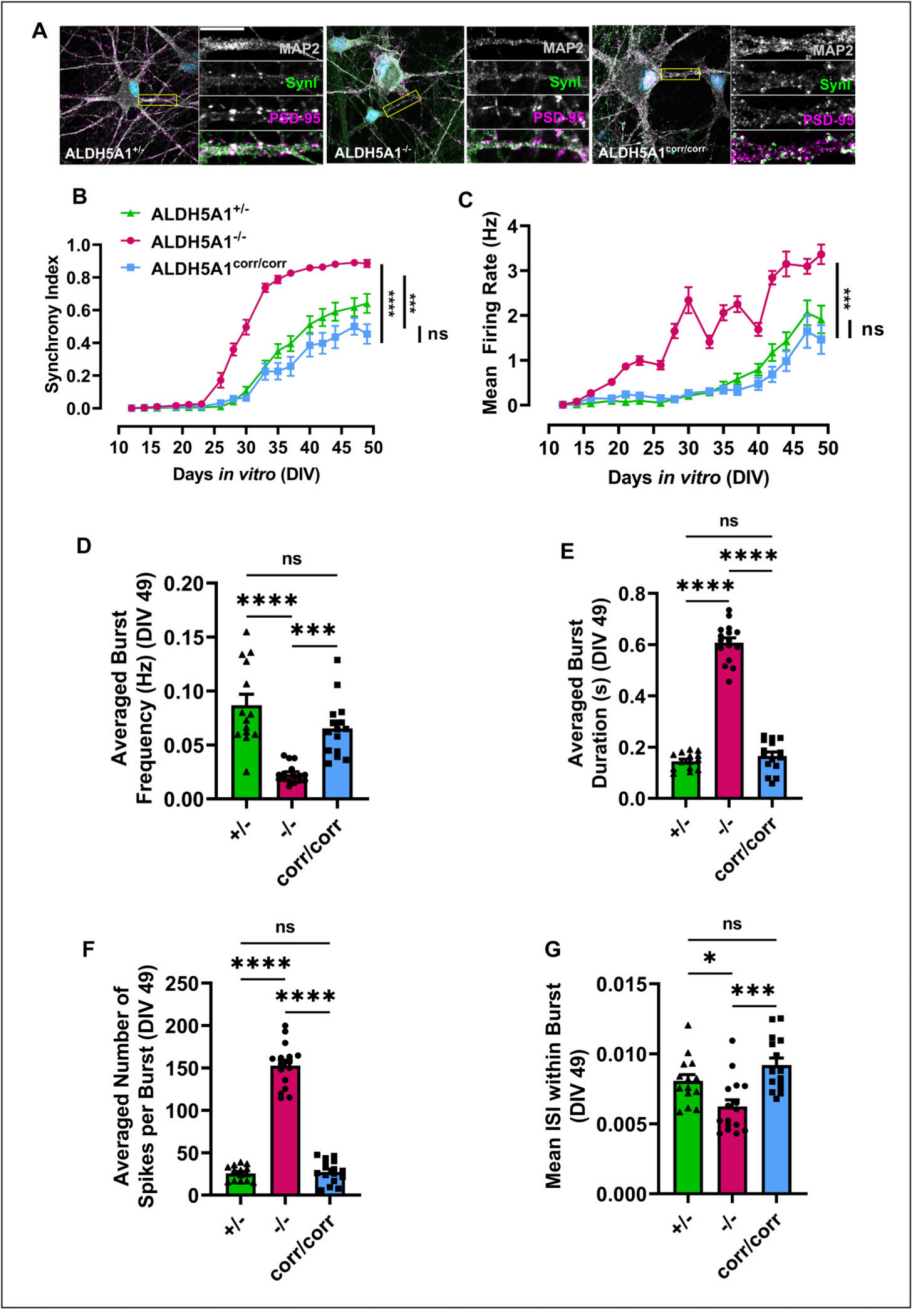
Characterization of the developing neuronal network in cultures of SSADH-deficient iNs. (A) Representative images of hiPSC-derived iNs from parental control (*ALDH5A1*^+/−^), patient (*ALDH5A1*^−/−^) and CRISPR corrected (*ALDH5A1*^corr/corr^) at DIV 50 labelled with MAP2 (gray), SYN I (green) and PSD95 (magenta) in co-culture with hiPSC-derived astrocytes. Scale bar 10 μm. Multi electrode array analysis shows (B) synchrony index and (C) mean firing rate of hiPSC-derived iNs from all genotypes in co-culture with hiPSC-derived astrocytes plotted over time (DIV10 up to DIV50). n = 3 independent differentiations; 16 independent wells per differentiation; two-way ANOVA with Tukey’s multiple comparisons test; F (32, 765) = 8.031; ****p < 0.0001; ***p < 0.001; ns = non-significant). We compiled bursting activity at DIV49 (D) average burst frequency F (2, 42) = 6.531 (E) averaged burst duration F (2, 42) = 2.743 (F) averaged number of spikes per burst F (2, 42) = 5.180 (G) mean inter spike interval (ISI) within burst F (2, 42) = 0.2319 (mean ± s.e.m.; n = 3 independent differentiations; 16 independent wells per differentiation; one-way ANOVA with Tukey’s multiple comparisons test; ****p < 0.0001; **p < 0.01; *p < 0.05; ns = non-significant). (For interpretation of the references to colour in this figure legend, the reader is referred to the web version of this article.)

To investigate the impact of SSADHD on glutamatergic neuronal network, we co-cultured them with commercially available wild-type hiPSC-derived astrocytes and analyzed their synaptic development using pre- and post-synaptic markers, synapsin-1 (SYN1) and post-synaptic density 95 (PSD-95), respectively. iNs expressed SYN1 and PSD-95 in close apposition at differentiation day 50 consistent with the formation of functional synapses (Fig. 3A). We observed expression of each of these synaptic markers in all genotypes, demonstrating that hiPSCs can differentiate into functionally mature neurons regardless of the allelic dosage of *ALDH5A1* under these conditions (Fig. 3A).

To characterize the activity of these mature neurons and their networks, we used two complementary approaches: multi-electrode array (MEA) to investigate the developing glutamatergic network and calcium imaging to measure functional activity in single cells within the network. MEA allows the extracellular recording of neuronal cultures through maturation. Epilepsy is characterized by hypersynchronous neuronal discharges (Rao and Lowenstein, 2015) and given the high prevalence of epilepsy in patients with SSADHD, we examined the synchrony index from each genotype, which is related to the cross- correlation between all pairs of electrodes within a well. We observed that the *ALDH5A1*^−/−^ neurons demonstrated earlier synchrony and significantly greater synchrony indices than *ALDH5A1*^+/−^ iNs (Fig. 3B and Fig. S4). Additionally, we observed that *ALDH5A1*^−/−^ neurons become more active earlier and displayed a significantly greater mean firing rate until day 50 compared to *ALDH5A1*^+/−^ (Fig. 3C). To further characterize the network formed by SSADH deficient iNs, we investigated the bursting activity as defined by alternating periods of high and low activity, which are a hallmark of functional networks and observed that *ALDH5A1*^−/−^ neurons were bursting less often than controls (Fig. 3D). Although the bursts occurred less frequently, bursts were both significantly longer (Fig. 3E and Fig. S4), and also consisted of more and faster spikes than controls (Fig. 3F, G and Fig. S4). These results suggest an altered activity in the network formed by SSADH-deficient iNs. Interestingly, the network activity, synchrony index, and bursting activity in the *ALDH5A1*^corr/corr^ iNs were similar to the *ALDH5A1*^+/−^ neurons, suggesting the changes observed in *ALDH5A1*^−/−^ are linked to the deficient SSADH enzyme and can be ameliorated by CRISPR correction (Fig. 3B to G).

**Fig. 4.**
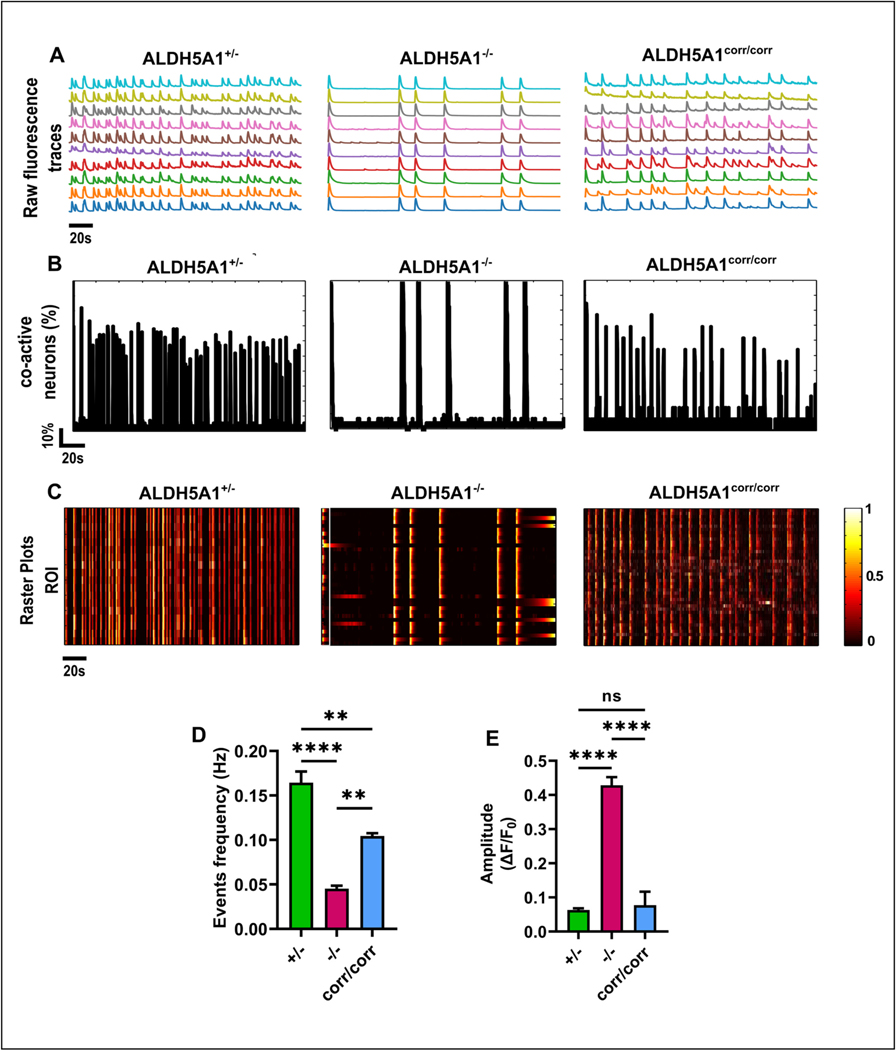
Characterization of neuronal activity in SSADH-deficient iNs with a single-cell level readout. (A) Representative raw traces of changes in fluorescence of jRCaMP1b as a measure of spontaneous neuronal activity in hiPSC-derived iNs from all three genotypes in co-culture with hiPSC-derived astrocytes at DIV 77. Traces of 10 representative neurons were recorded simultaneously in the same field of view for 200 s (scale 20s). (B) Percentage of co-active cells during each calcium event in the recording used for (a) in all three genotypes over time (scales 10% and 20s). (C) Colored raster plot for the recordings used in (a) for all three genotypes (scale 20s). Characterization of neuronal activity (D) events frequency F (2, 93) = 10.80, (E) amplitude of events, F (2, 250) = 60.21.

Additionally, we transduced iNs in co-culture with hiPSC-derived astrocytes using a lentiviral expression vector under a human synapsin promoter (pLV-hSyn-jRCaMP1b) at DIV21 and consistently observed expression of jRCaMP1b one-week post-transduction and performed recordings of neuronal activity of mature neurons at DIV77. We extracted the changes of fluorescence recorded over time (200 s) and plotted the raw traces for ten neurons recorded simultaneously (Fig. 4A). We used both NeuroCa (Jang and Nam, 2015) and FluoroSNNAP (Patel et al., 2015) software for analysis of the network with single cell resolution, including bursting activity, with the percentage of co-active cells characterized by the number of active regions of interest (ROIs) detected at each frame normalized to the total number of ROI detected in the field of view. Using this approach, we observed that SSADH-deficient neurons reached a maximum of 100% co-active cells, whereas controls reached a maximum of 70 and 80% (Fig. 4B). Additionally, the raster plots show that the detected bursts were longer in SSADHD, confirming the MEA findings (Fig. 4C and Fig. 3). Taking advantage of the single-cell readout that calcium imaging offers, we observed that calcium events occurred less often in the *ALDH5 A1*^−/−^ compared to *ALDH5 A1*^+/−^ and *ALDH5 A1*^corr/corr^ (Fig. 4D). Although the neurons were active less often, the averaged amplitude of the events was four-fold higher in the *ALDH5 A1*^−/−^ compared to *ALDH5 A1*^+/−^ and *ALDH5 A1*^corr/corr^ (Fig. 4E). Importantly, the activity in the *ALDH5 A1*^corr/corr^ iNs was similar to the *ALDH5 A1*^+/−^, suggesting that the changes of neuronal activity are linked to *ALDH5A1* mutation and the deficient SSADH enzyme (Fig. 4D and E).

### 4.5. SSADH-deficient iGABA neurons show down-regulation of genes associated with GABAergic synaptic transmission

**Fig. 5.**
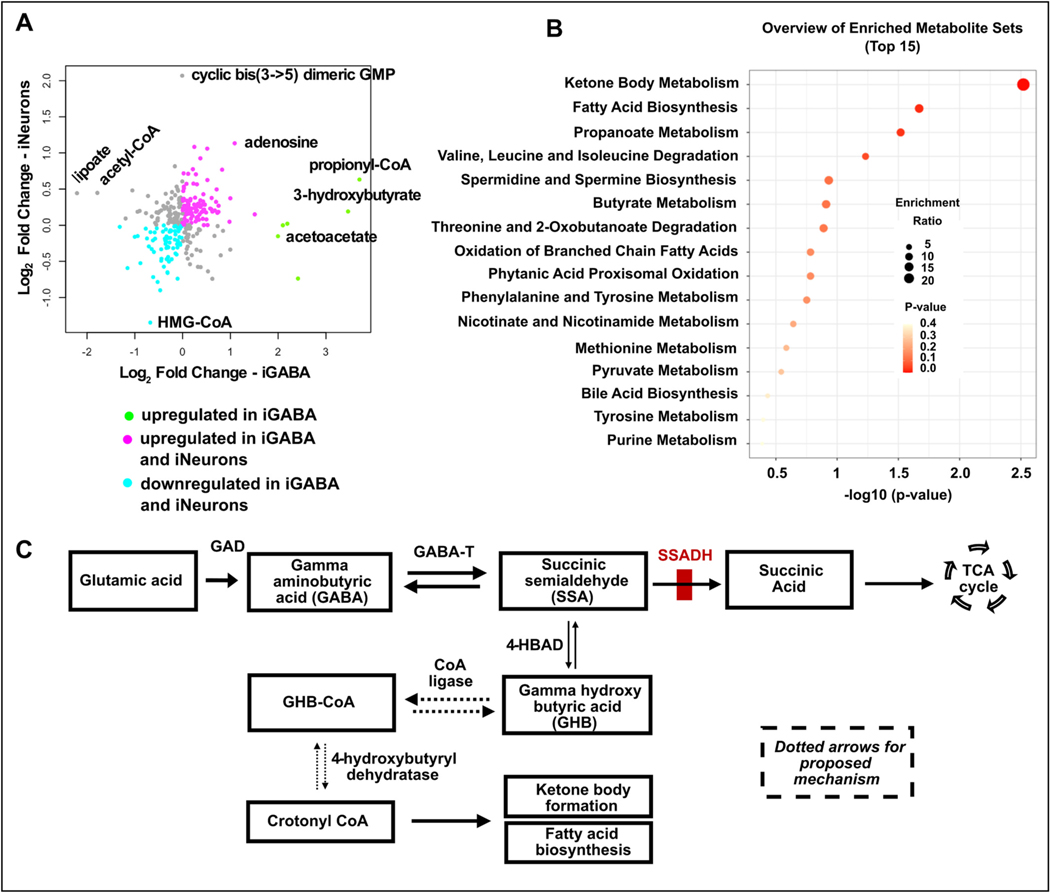
Metabolites changes associated with SSADHD in iNs and iGABA. (A) Scatterplot of metabolomic data from iN and iGABA neurons. The log2 fold change of each metabolite between proband and control in iGABA and iN is shown on the x-axis and y-axis, respectfully. The identity of specific metabolites with substantial changes are displayed on the plot. Most metabolites show similar changes between iN and iGABA neurons, either up-regulated in both neuronal subtypes (magenta) or down-regulated in both neuronal subtypes (cyan). However, there was a small group of metabolites that showed substantial increase in iGABA neurons without an associated change in iNs (green). (B) Overview of the top 15 upregulated enriched metabolite sets in iGABA *ALDH5A1*^−/−^ neurons compared to control from all families at DIV 35 with the two most significant sets related to ketone body metabolism and fatty acid biosynthesis (n = 3, *p* values as indicated). (C) GABA metabolism pathway where the block in SSADHD is indicated in red. Dotted arrows show a proposed mechanism for the increased ketone body formation and fatty acid biosynthesis observed in iGABA *ALDH5A1*^−/−^ compared to control. (For interpretation of the references to colour in this figure legend, the reader is referred to the web version of this article.)

Having established a robust human *in vitro* model of SSADHD, we next sought to investigate pathways altered in both subtype of neurons using unbiased metabolomic profiling. To do so, we performed metabolomic analyses of the iGABA and iNeurons using liquid chromatography-mass spectrometry (LC-MS) (Yuan et al., 2012). Following hierarchical cluster analysis (Fig. 5A), Metabolite Set Enrichment Analysis (MSEA) was performed on detected metabolites to identify which pathways were affected by the specific metabolites. In parallel, we also used the Metabolomic Pathway Analysis (MetPA) module of MetaboAnalyst, which combines results from the pathway enrichment analysis with the pathway topology analysis (Fig. 5B). We found that nearly all changes in metabolites found in iNs were also observed in iGABA neurons (Fig. 5A, cyan and magenta). In contrast, we identified significant changes specific to iGABA neurons (Fig. 5A, green). A graphical list of the pathways identified, and their relative impacts are shown in (Fig. 5B). Top pathways were related to ketone body metabolism and fatty acid biosynthesis, suggesting the existence of an alternative glutamate/GHB degradation pathway whereby the dehydration of GHB-CoA to crotonyl-CoA increases fatty acid biosynthesis and ketone body formation (Fig. 5C). Consistent with this hypothesis, levels of acetoacetate and 3-hydroxybutyrate were significantly increased (Fig. 5A).

**Fig. 6.**
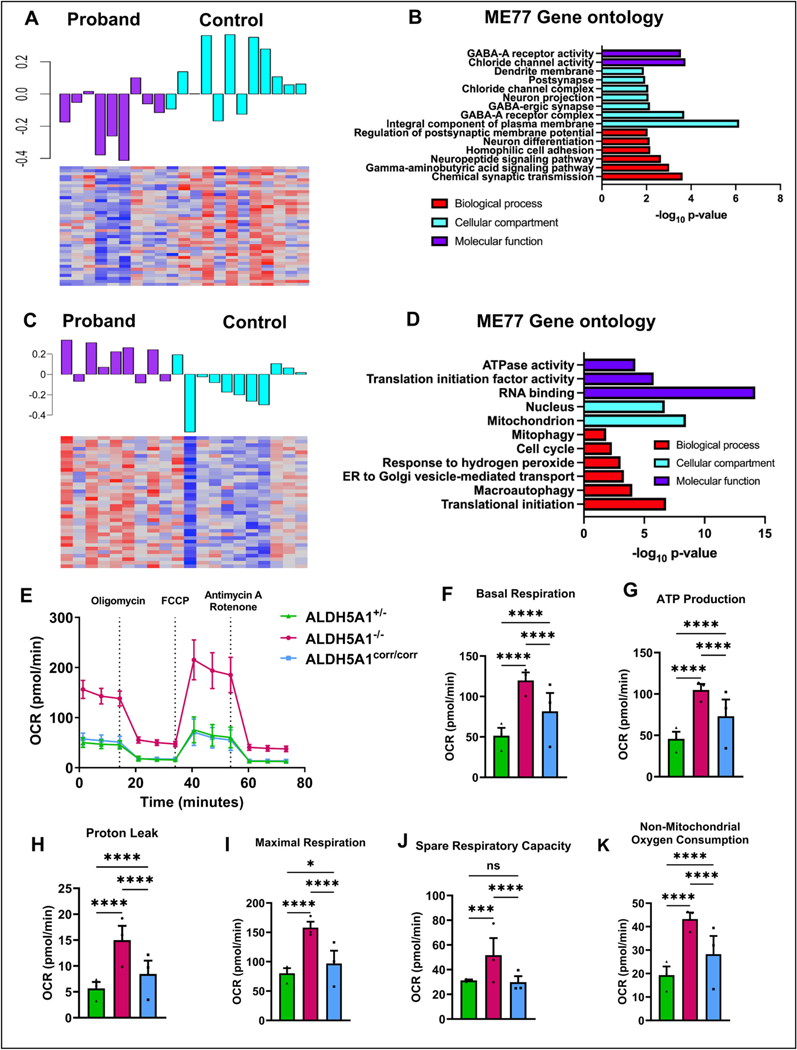
Gene expression changes associated with SSADHD and seahorse assay. (A) Co-expression module ME77 contains genes down-regulated in SSADH deficient iGABA neurons. The barplot shows the first principal component of gene expression within module or the Module Eigengene, where the cyan is control and the magenta is proband. The heatmap represents the normalized expression of all genes within the module with red denoting higher expression and blue lower expression. (B) The barplot shows the -log_10_ *p*-value of the enrichment of genes within the ME77 module using Gene Ontology. (C) Co-expression module ME54 contains genes up-regulated in SSADH deficient iNs. The barplot shows the first principal component of gene expression within a module or the Module Eigengene, where the cyan is the control and the magenta is the proband. The heatmap represents the normalized expression of all genes within the module, with red denoting higher expression and blue lower expression. n = 3 independent differentiations. (D) The barplot shows the -log_10_ p-value of the enrichment of genes within the ME54 module using Gene Ontology. (E-K) Seahorse assay performed on iNeurons parental control (*ALDH5A1*^+/−^), patient (*ALDH5A1*^−/−^) and (*ALDH5A1*^corr/corr^); (mean ± s.e.m.; n = 3 independent differentiations, 20 replicates/genotype for each differentiation; two-way ANOVA with Tukey’s multiple comparisons test; ****p < 0.0001; ***p < 0.001; **p < 0.01; *p < 0.05; ns = non-significant) (E) Mitochondrial oxygen consumption rate (OCR) in pmol/min (F) Basal respiration; Interaction F (4, 171) = 52.98; Row Factor F (2, 171) = 444.3; Column Factor F (2, 171) = 905.8 (G) ATP Production; Interaction F (4, 171) = 53.58; Row Factor F (2, 171) = 388.0; Column Factor F (2, 171) = 810.2 (H) Proton leak; Interaction F (4, 171) = 17.74; Row Factor F (2, 171) = 17.6; Column Factor F (2, 171) = 310.7 (I) Maximal Respiration; Interaction F (4, 171) = 9.995; Row Factor F (2, 171) = 6.750; Column Factor F (2, 171) = 68.74 (J) Spare respiratory capacity Interaction F (4, 171) = 5.784; Row Factor F (2, 171) = 4.841; Column Factor F (2, 171) = 11.40 (K) Non-mitochondrial oxygen consumption Interaction F (4, 171) = 19.90; Row Factor F (2, 171) = 180.2; Column Factor F (2, 171) = 393.3. (For interpretation of the references to colour in this figure legend, the reader is referred to the web version of this article.)

To examine the alterations in molecular pathways that accompany the loss of *ALDH5A1*, we performed RNA sequencing in iGABA neurons and used Weighted Gene Co-expression Network Analysis (WGCNA). The co-expression pattern within iGABA neurons that best corresponded to genotype (ME77) consisted of down-regulated genes in *ALDH5A1*^−/−^ iGABA neurons (Fig. 6A). Using gene ontology analysis, we observed that these down-regulated genes were highly enriched in categories associated with GABAergic synaptic transmission (Fig. 6B). For example, the GABA receptor subunits GABRA4, GABRA2, and GABRG1 demonstrated high connectivity within this group of genes (Fig. S5A). To investigate whether this group of genes might be associated with neurodevelopmental symptoms frequently associated with SSADHD such as ASD, we studied the relationship of this co-expression pattern with genes that have been implicated in ASD. Remarkably, we found that ASD candidate genes from SFARI database demonstrated opposite regulation compared to the genes in ME77 or up-regulation in SSADH deficient hiPSC-derived iGABA neurons (Fig. S5B). Together, these data show that genes associated with GABAergic neurotransmission are down-regulated and that ASD candidate genes are up-regulated in inhibitory SSADH deficient neurons.

### 4.6. SSADH-deficient iNs show changes in genes associated with mitochondria and mitochondria function

To examine the effect of loss of *ALDH5A1* in iNs, we analyzed gene expression patterns in these cells. We first asked whether the genes that were altered in iGABA SSADH deficient neurons (ME77) were also affected in iNs, but we found that these changes were specific to iGABA neurons and not observed in iNs (Fig. S5C). We hypothesized that these changes might be specific to iGABA neurons because genes involved in GABAergic synaptic transmission might be expressed at higher levels in iGABA neurons at baseline compared to iNs. Indeed, we found that genes in the ME77 co-expression module showed increased expression in control iGABA neurons compared to control iNs (Fig. S5D). We then performed WGCNA on the iNs samples to investigate the effect of *ALDH5A1* deficiency on these neurons. The co-expression pattern that showed the strongest correspondence to genotype contained genes that were up-regulated in *ALDH5A1*^−/−^ iNs (Fig. 6C). Functional annotation on this group of genes revealed significant enrichment in genes associated with mitochondrial function, including the mitochondrial ribosome (Fig. 6D). Additionally, we compared the changes in the genes associated with mitochondria in both neuronal subtypes and found that these genes were specifically altered in iNs with no trend towards alteration in iGABA neurons (Fig. S5E). We then examined the expression levels of these genes in control iGABA and iNs, and surprisingly, we observed that these genes were expressed at similar levels in these two neuronal cell subtypes (Fig. S5F). Taken together, these data demonstrate that genes associated with mitochondrial function are preferentially altered in *ALDH5A1*^−/−^ glutamatergic neurons, but not in GABAergic neurons.

To further investigate the effect of SSADH deficiency on mitochondria function, we measured oxygen consumption rates (OCR) in iNs (Fig. 6E to K). Remarkably, we found dramatic changes in mitochondrial function in *ALDH5A1*^−*/*−^ iNs. Initially, we observed significantly increased baseline respiration (Fig. 6F) and ATP production (Fig. 6G) suggesting an increase in ATP demand. After inhibition of ATP synthase by oligomycin, we observed significantly increased proton leak in *ALDH5A1*^−*/*−^ iNs (Fig. 6H), which could be due to several factors including increased UCP (uncoupling protein) activity, damage to the inner mitochondrial membrane and/or ETC (electron transport chain) complexes. We then treated iNs with an uncoupler, FCCP (carbonyl cyanide *p*-trifluoromethoxyphenylhydrazone), to measure maximal respiration (Fig. 6I) and spare respiration (Fig. 6J), and we found that these parameters were both significantly elevated in *ALDH5A1*^−*/*−^ iNs. Finally, with the addition of antimycin A and rotenone, we observed elevated non-mitochondrial oxygen consumption (Fig. 6K), which has been shown to be linked with the presence of stressors including reactive oxygen species (Hill et al., 2012). Taken together, genes associated with mitochondria were upregulated in a subtype specific manner, and iNs display altered mitochondrial function in SSADHD.

## 5. Discussion

Here, we used stem cell-derived neurons to probe the role of SSADHD in human inhibitory and excitatory cortical neurons. This approach allowed us to study these cell types independently and disambiguate the roles of *ALDH5A1* in a cell type specific manner. As expected, *ALDH5A1*^−/−^ iGABA neurons displayed increased GHB levels and changes in metabolites related to the ketone body metabolism and fatty acid biosynthesis. Furthermore, we found that the biallelic correction using CRISPR or re-expression of *ALDH5A1* using mRNA transfection restored not only the production of SSADH but also its function, resulting in similar GHB levels to the iGABA neurons from the heterozygous parental control (*ALDH5A1*^+/−^). Strikingly, we also identified robust phenotypic differences in the iNs and found that *ALDH5A1*^−/−^ iNs displayed increased neuronal activity and greater synchrony. In addition, *ALDH5A1*^−/−^ iNs had altered expression in genes associated with mitochondrial function, as well as dramatic differences in oxygen consumption. These data demonstrate that the pathophysiology of SSADHD extends beyond differences in GABA levels and implicate mitochondrial dysregulation in excitatory neurons as a critical molecular abnormality.

Consistent with data from patient brain (Gibson et al., 1998), we observed a 30-fold increase of GHB and 2- to 4-fold increase of GABA levelts in *ALDH5A1^−/−^*iGABA neurons. *ALDH5A1*^−*/*−^ animals have significantly increased GABA during embryonic development, display markedly smaller brains with the cerebellum particularly affected, and are characterized by ataxia and an epileptic transition from absence seizures to ultimately fatal convulsive status epilepticus by three weeks of age (Cortez et al., 2004; Jansen et al., 2008). We also found increased levels of metabolites related to ketone body metabolism and fatty acid biosynthesis in *ALDHA5A1*^−*/*−^ iGABA neurons, which we suspect is due to an alternative glutamate/GHB degradation pathway involving the dehydration of GHB-CoA to crotonyl CoA. However, it has been difficult to reconcile hyperactivity with increased levels of inhibitory neurotransmitters. Several studies have demonstrated downregulation of GABA receptors (Walters et al., 2021; Gibson et al., 2005b). In fact, GHB itself may contribute to the downregulation of presynaptic and post-synaptic GABA receptor expression (Gibson et al., 2005a). We also observed downregulation of genes associated with GABAergic synaptic transmission in human iGABA neurons. Interestingly, GABRA4 was highly connected within this group of genes, and receptors containing this subunit have been shown to bind to GHB (Absalom et al., 2012). One potential explanation of these data is that the ME77 co-expression pattern represents a compensatory down-regulation of GABAergic genes in response to high levels of GHB. This would suggest that alterations in GABAergic receptors are a homeostatic response to inhibitory transmitter levels and less likely to be causing insufficient inhibition and thus hyperactivity.

Few studies have examined the effects of loss of *ALDH5A1* on excitatory neurons, but we found that these neurons displayed altered activity and increased synchrony. Prior studies have suggested increased levels of glutamate in Aldh5a1 animals (Gupta et al., 2004). However, we did not observe any significant changes in glutamate, GABA, or GHB, demonstrating that *ALDH5A1* is involved in critical processes beyond GABA catabolism in excitatory neurons. Gene expression studies demonstrated dysregulation of genes associated with mitochondrial function, including the mitochondrial ribosome, and that these genes were specifically altered in iNs. Consistent with this finding, other studies in non-neuronal cells have suggested that ALDH5A1 is localized to the mitochondria and plays a role in its function (Menduti et al., 2020). Additionally, we observed dramatic alterations in mitochondrial function in *ALDH5A1*^−/−^ iNs, suggesting myriad metabolic changes such as increases in ATP demand, UCP activity, damage to the inner mitochondrial membrane and/or ETC complexes and the presence of stressors including reactive oxygen species. These data demonstrate that loss of *ALDH5A1* in excitatory human neurons causes intrinsic changes to excitability and suggest that mitochondrial function is significantly altered in these cells. In neurons, mitochondria play additional important roles in calcium homeostasis, control of membrane excitability and neurotransmission and plasticity (Norat et al., 2020; Rose et al., 2017). Additionally, neurons displaying altered excitability are often accompanied by sustained higher cytosolic calcium levels leading to mitochondrial calcium overload, which in turn increases mitochondrial ROS production and mitochondrial damage (Verma et al., 2022). Given the multifaceted functions of mitochondria (Swann and Rho, 2014; Collier et al., 2023; Ebrahimi-Fakhari et al., 2016), this abnormality could contribute to the development of neurological symptoms in SSADHD.

Taken together, our data generated using hiPSC-derived neurons provide unique insights into the cellular and molecular basis of SSADHD. We observe expected changes in GABA metabolism, as well as downstream consequences at the gene expression and metabolomic levels. However, these effects appear to be primarily a compensatory response to abnormal neurotransmitter levels. In contrast, excitatory neurons display altered activity and hypersynchrony, as well as mitochondrial dysfunction. These data highlight a potential new direction to unravel the pathophysiology of SSADHD, which could lead to the development of novel therapeutic strategies.

## Supplementary Material

1

## Figures and Tables

**Table 1:** Summary of clinical features and human iPSCs lines from SSADH deficient patients, sex-matched unaffected parental controls and CRISPR corrected lines.

Family	Severity	Genotype	Sex	Participant	Genetic variant (NM_170740.1)	Domain

001	Severe with seizures	*ALDH5A1^−/−^*	F	Proband	c.1226G > A / c.1226G > A; p.Gly409Asp	Catalytic Domain
	−	*ALDH5A1^+/−^*	F	Sex-matched parent	c.1226G > A / c.1226G	
	−	*ALDH5A1* ^corr/corr^	F	CRISPR edited	c.1226G / c.1226G	
002	Mild no seizure	*ALDH5A1^−/−^*	M	Proband	c.612G > A / c.612G > A; p.Trp204Ter	NAD^+^ Binding Domain
	−	*ALDH5A1^+/−^*	M	Sex-matched parent	c.612G > A / c.612G	
003	Mild no seizure Abnormal EEG	*ALDH5A1^−/−^*	M	Proband	exon 4 c.612G > A; pTrp204* exon 9 c.1273C > T; p.Arg245* (R412X)	NAD^+^ Binding and catalytic domains
	−	*ALDH5A1^+/−^*	M	Sex-matched parent	exon 4 c.612G > A; pTrp204* Exon 9 c.1273C	

**2.1.** Key resources table

REAGENT or RESOURCE	SOURCE	IDENTIFIER

Antibodies		
Rabbit anti-GABA	Sigma-Aldrich	Cat#A2052; RRID: AB_477652
Chicken anti-MAP2	Abcam	Cat#ab5392; RRID: AB_2138153
Rabbit anti-TBR1	Abcam	Cat#ab31940; RRID: AB_2200219
Alexa Fluor^™^ 488 goat anti-chicken IgG (H + L)	Invitrogen	Cat#A11039; RRID: AB_142924
Alexa Fluor^™^ 647 goat anti-rabbit IgG (H + L)	Invitrogen	Cat#A21244; RRID: AB_2535812
Hoechst	Invitrogen	Cat#H3569; RRID: AB_2651133
Bacterial and virus strains		
pLV-TetO-hNGN2-P2A-mCherry-T2A-Puro	This paper	N/A
pLV-TetO-hDLX2-P2A-hASCL1-T2A-Puro	This paper	N/A
pLV-hSyn-jRCaMP1b	This paper	N/A
Biological samples		
Human iPSCs (Table 1)	This paper	N/A
Chemicals, peptides, and recombinant proteins		
ALDH5A1 mRNA	TriLink	Cat#ENST00000357578.8; TriLink, 1 mg scale, 5-Methoxyuridine, 1 mM Sodium Citrate, pH 6.4
mRNA CleanCap^®^ EGFP mRNA Media	TriLink	Cat#L-7601
Lipofectamine MessengerMAX mRNA Transfection	Invitrogen	Cat#LMRNA001
B27 Supplement	Life Technologies	Cat#17504–044
N2 Supplement	Thermo Fisher	Cat#17502048
Poly-D-lysine hydrobromide	Sigma-Aldrich	Cat#P6407
Laminin	Life Technologies	Cat#23017–015
Y-27632	Cayman	Cat#10005583
Doxycycline	Millipore	Cat#324385–1GM
Puromycin	Invitrogen	Cat#ant-pr-1
BDNF	Peprotech	Cat#450–02
GDNF	Peprotech	Cat#450–10
NT3	Peprotech	Cat#450–03
Papain	Worthington	Cat##LK003178
DNAseI	Worthington	Cat#LK003172
Critical commercial assays		
GHB kit	BUHLMANN Diagnostics Corp, Amherst, NH	#CatKK-GHB-U
GABA kit	LDN, Nordhorn	#CatBA E-2500
Seahorse XF Cell Mito stress	Agilent	Cat# 103015–100
CytoView MEA plates	Axion Biosystems	#CatM768-tMEA-48B
Experimental models: Cell lines		
Human iPSC-derived astrocytes	NCardia	Cat#M0605
Oligonucleotides		
Mycoplasma testing	CReM Boston	N/A
Myco280_CReM (5'- ACACCATGGGAGYTGGTAAT-3'); Myco279_CReM (5'- TTCWTCGACTTYCAGACCCAAGGCAT- 3') MGSO-5 (5'-TGCACCATCTGTCACTCYGTTAACCTC- 3') GPO-3 (5'- GGGAGCAAACAGGATTAGATACCCT- 3')	University	
Recombinant DNA		
pLV-TetO-hNGN2-P2A-mCherry-T2A-Puro	This paper	N/A
pLV-TetO-hDLX2-P2A-hASCL1-T2A-Puro	This paper	N/A
pLV-hSyn-jRCaMP1b	This paper	N/A
Software and algorithms		
Fiji	Schindelin et al. (Schindelin et al., 2012)	https://imagej.net/software/fiji/
FluoroSNNAP	Patel et al. (Patel et al., 2015)	https://www.seas.upenn.edu/~molneuro/software.html
Nikon NIS Elements		https://www.microscope.healthcare.nikon.com/products/software/nis-elements
Gene ontology analysis	DAVID	https://david.ncifcrf.gov/summary.jsp

## Data Availability

• All data reported in this paper will be shared by the lead contact by request. • This paper does not report original code. • Any additional information required to reanalyze the data reported in this paper is available from the lead contact upon request.

## Data availability

Data and resources generated in this study are available upon reasonable request.

## Abbreviations:

ASD: Autism Spectrum Disorder
CNS: Central Nervous System
DIV: Days *in vitro*
GABA: γ-amino butyric acid
GECIs: Genetically Encoded Calcium Indicators
GHB: γ-hydroxybutyrate
hiPSCs: Human Induced Pluripotent Stem Cells
iGABA: GABAergic neurons
iNs: Glutamatergic neurons
LC-MS: Liquid Chromatography-Mass Spectrometry
MetPA: Metabolomic Pathway Analysis
MSEA: Metabolite Set Enrichment Analysis
OCR: Oxygen Consumption Rate
PSD-95: Postsynaptic Density 95
ROI: Region of Interest
SSADHD: Succinic semialdehyde dehydrogenase deficiency
SUDEP: Sudden Unexpected Death in Epilepsy
SYN1: Synapsin-1
UCP: Uncoupling Protein
WGCNA: Co-expression Network Analysis
