# Supplementary material for: ALDH5A1-deficient iPSC-derived excitatory and inhibitory neurons display cell type specific alterations
Source: Neurobiol Dis. Author manuscript; Available in PMC 2024 Feb 5. (PMC10843729; doi:10.1016/j.nbd.2023.106386)
Supplement: 1 [file NIHMS1956818-supplement-1.pdf]

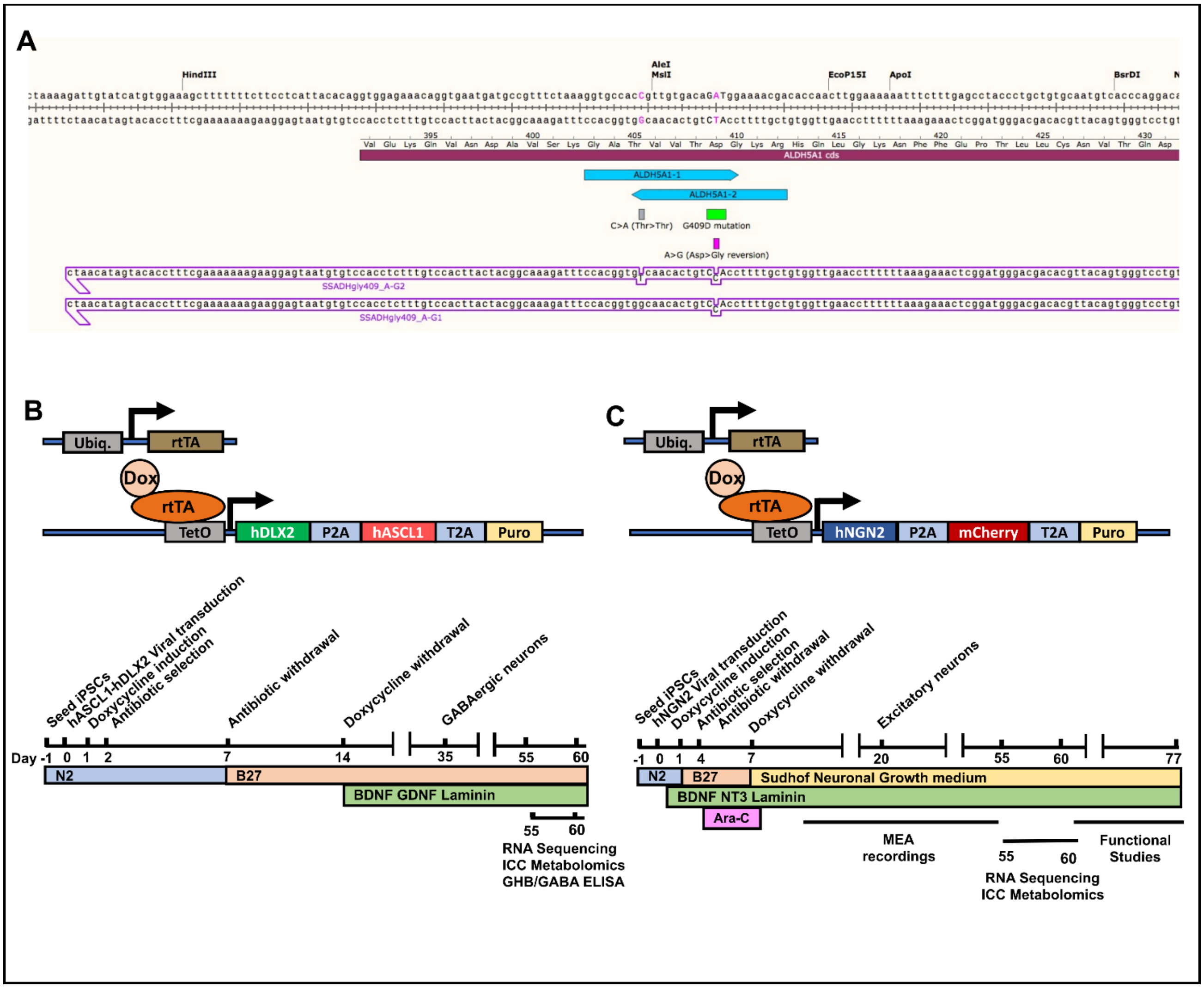

Supplemental Figure S1. Generation of CRISPR-corrected hiPSC line and differentiation methods to generate iGABA and iNs.

(A) Summary of clinical features and human iPSCs lines from SSADH deficient patients, sex-matched unaffected parental controls and CRISPR corrected lines. **(B)** SSADH Asp409Gly reversion using CRISPR and ssODN to generate *ALDH5A1*$^{corr/corr}$. **(C)** Design of construct and schematic presentation of the iGABA neurons differentiation protocol and assays. **(D)** Design of construct and schematic presentation of the iNs differentiation protocol and assays.

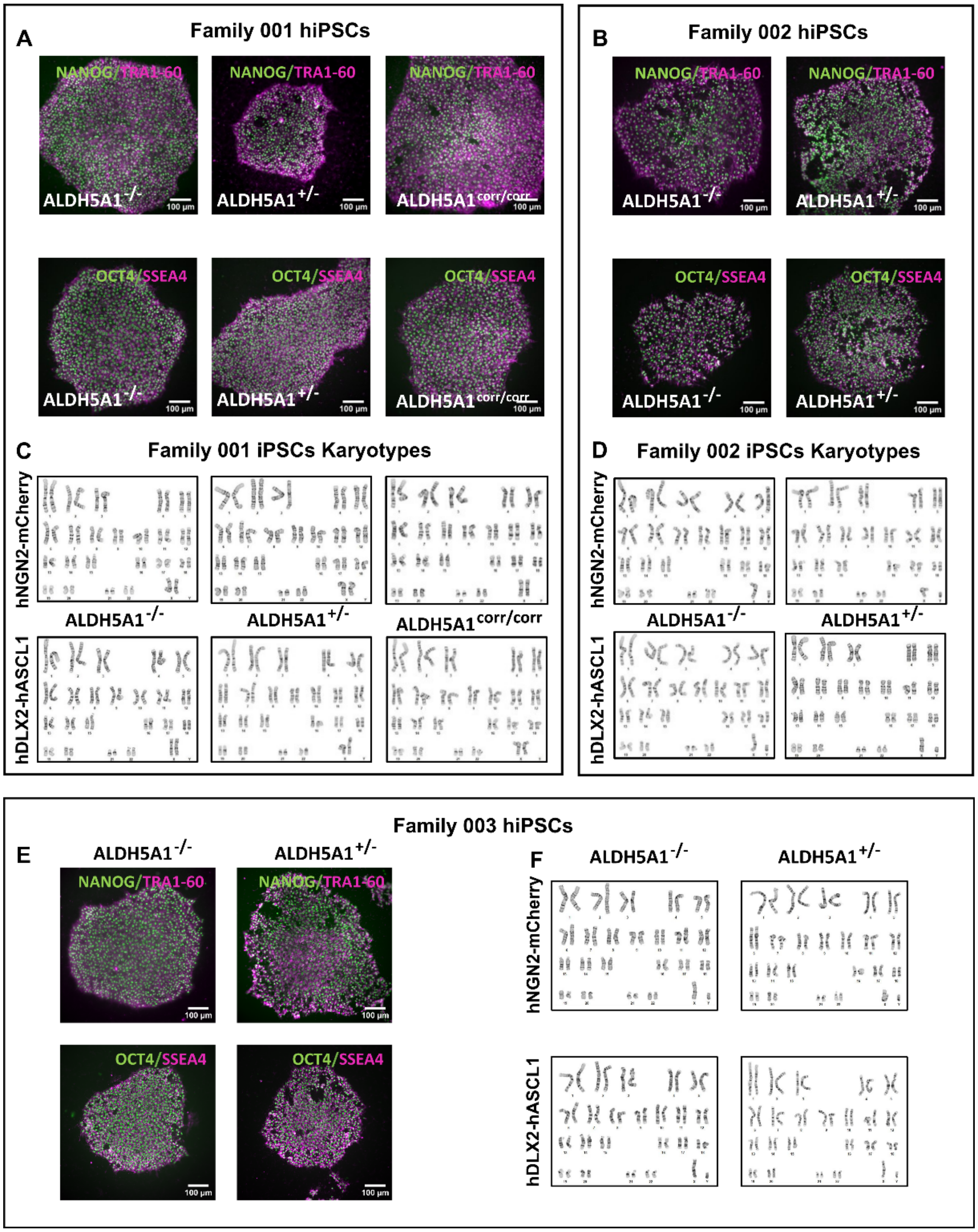

Figure S2. Generation and characterization of the hiPSCs used in this study

(A,B,E) Successful reprogramming by characterizing the expression of pluripotency markers. Top panel: NANOG (Nanog homeobox x in green) and TRA-1-60 (podocalyxin in magenta). Bottom panel: OCT4 (octamer binding transcription factor 4 in green), SOX2 (SRY-Box Transcription Factor 2 in magenta), in undifferentiated pluripotent hiPSC colonies. **(C,D,F)** G-banded karyotype post-transduction with pLV-TetO-hNGN2-P2A-mCherry-T2A-Puro (hNGN2-mCherry) or pLV-TetO-hDLX2-P2A-hASCL1-T2A-Puro (hDLX2-hASCL1) showing a normal karyotype for all lines.

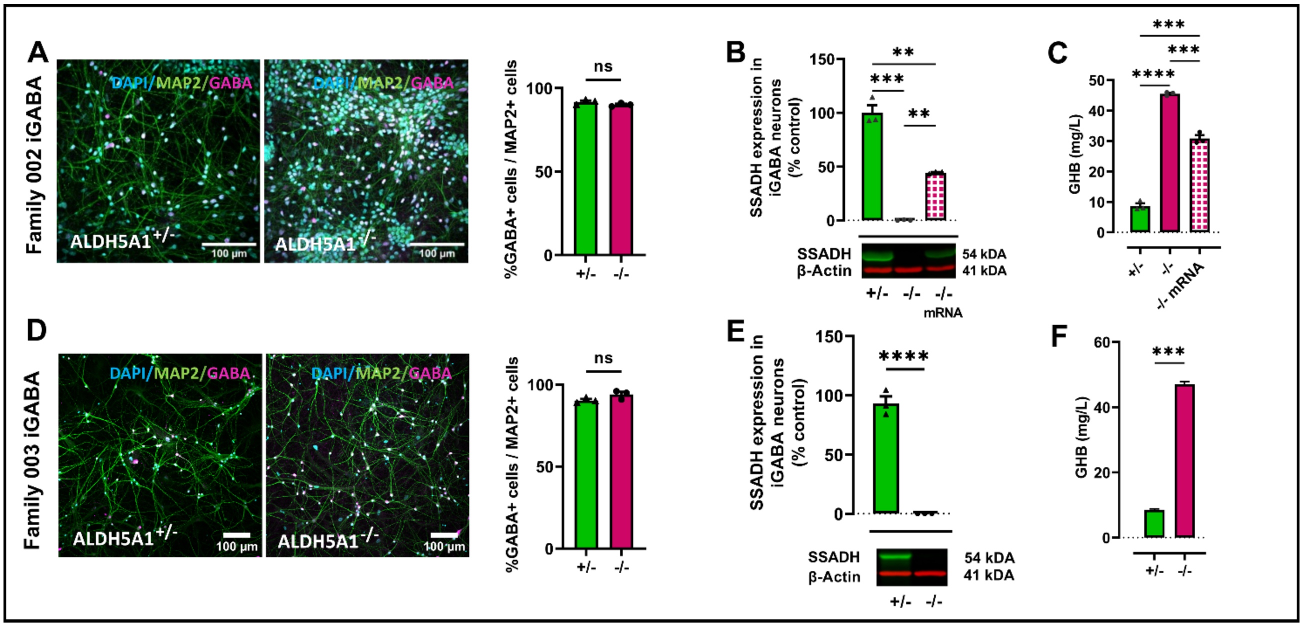

Figure S3. Characterization of iGABA neurons from Family 002 and 003

(A) Representative images of iGABA neurons from family 002 parental control ($ALDH5A1^{+/-}$) and patient ($ALDH5A1^{-/-}$) at DIV 55 in co-culture with hiPSC-derived astrocytes labeled with DAPI (cyan), MAP2 (green), GABA (magenta). Scale bar 100µm and quantification of GABA-positive neurons over MAP2 positive neurons showing no significant difference between all three genotypes used in this study (n=3). **(B)** Western blot of SSADH expression from family 002 iGABA neurons from all genotypes and SSADH-deficient neurons treated for 72hours at DIV 35. Quantification of SSADH levels from three separate transfections displayed as percentage of parental control ($ALDH5A1^{+/-}$) (mean ± s.e.m. values; n=3; ***$p < 0.001$, **$p < 0.01$; two-way ANOVA with Tukey's multiple comparisons test; $F(2, 4) = 132.1$). **(C)** ELISA quantification of GHB in family 002 iGABA neurons at DIV 35 and after 72hours treatment with mRNA (-/- treated) reveals significant increase in $ALDH5A1^{-/-}$ neurons and decrease following mRNA treatment (mean ± s.e.m. values; n=3 separate transfections; ****$p < 0.0001$; ***$p < 0.001$; ns=non-significant; two-way ANOVA with Tukey's multiple comparisons test; $F(2, 4) = 409.6$). **(D)** Representative images of iGABA neurons from family 003 parental control ($ALDH5A1^{+/-}$) and patient ($ALDH5A1^{-/-}$) at DIV 55 in co-culture with hiPSC-derived astrocytes labeled with DAPI (cyan), MAP2 (green), GABA (magenta). Scale bar 100µm and quantification of GABA-positive neurons over MAP2 positive neurons showing no significant difference between all three genotypes used in this study (n=3). **(E)** Western blot of SSADH expression from family 003 iGABA neurons from all genotypes at DIV 35. Quantification of SSADH levels from three separate differentiations displayed as percentage of parental control ($ALDH5A1^{+/-}$) (mean ± s.e.m. values; n=3; ****$p < 0.0001$, unpaired t-test, two-tailed; t=15.59, df=4). **(F)** ELISA quantification of GHB in family 003 iGABA neurons at DIV 35 showing significant increase in $ALDH5A1^{-/-}$ (n=3 independent differentiations; ***$p < 0.001$, unpaired t test; two-tailed; t=42.06, df=2).

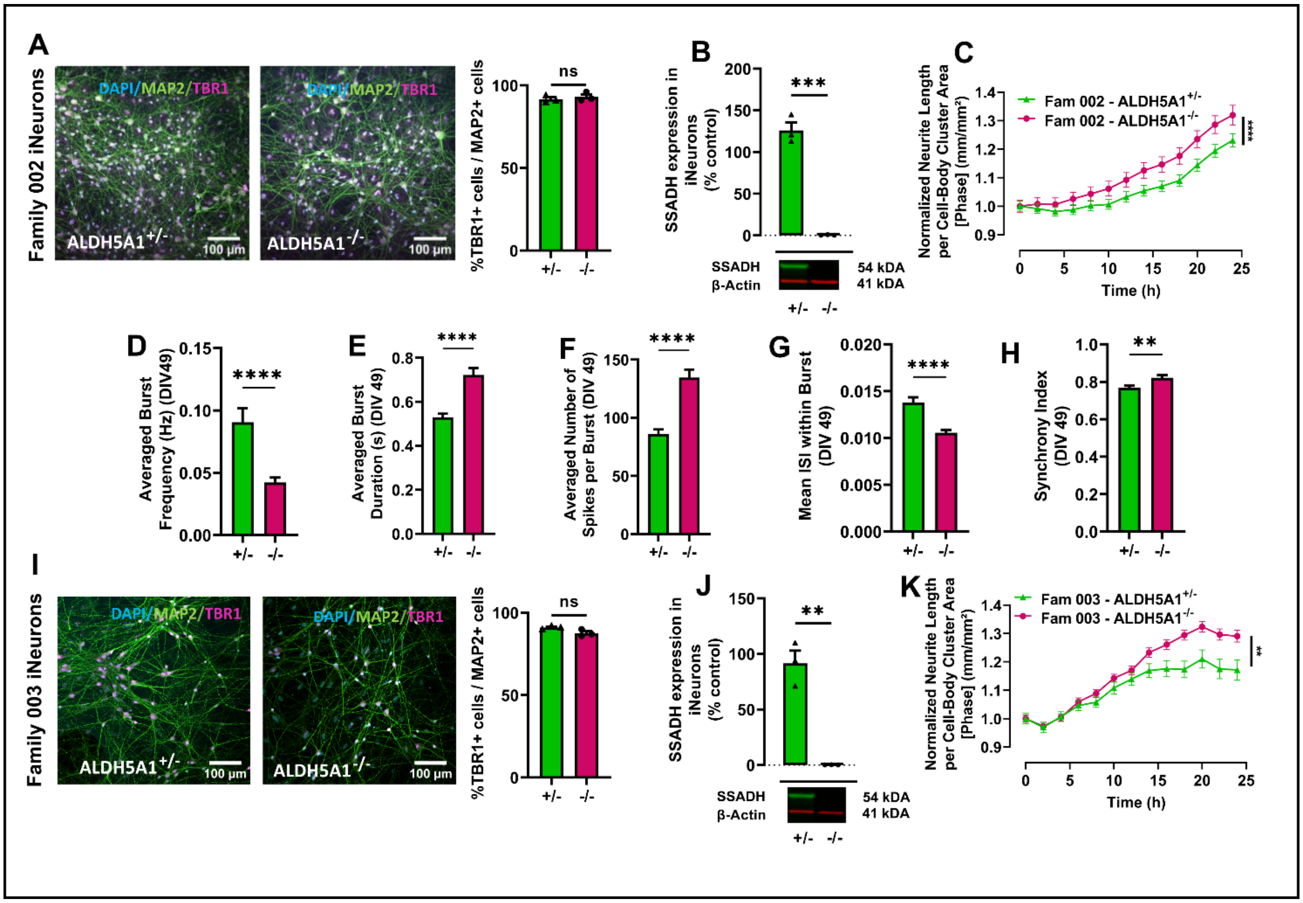

Figure S4. Characterization of iNs from Family 002 and 003

(A) Representative images of hiPSC-derived iNs from family 002 parental control (*ALDH5A1*$^{+/-}$) and patient (*ALDH5A1*$^{-/-}$) at DIV 55 in co-culture with hiPSC-derived astrocytes labeled with DAPI (cyan), MAP2 (green) and TBR1 (magenta). Scale bar 100µm. Quantification of TBR1-positive neurons over MAP2-positive neurons showed no significant difference between all three genotypes used in this study (mean ± s.e.m. values; n=3; ns=non-significant). **(B)** Western blot of SSADH expression from family 002 iNs DIV 30. Quantification of SSADH levels from three separate differentiations normalized to β-actin and displayed as a percentage of parental control (*ALDH5A1*$^{+/-}$) (mean ± s.e.m. values; n=3; ***$p < 0.001$; unpaired t test; two-tailed; t=13.04, df=4). **(C)** Normalized family 002 iNs neurite length per cell-body cluster area [phase] (mm/mm^2) demonstrates significantly longer neurites in *ALDH5A1*$^{-/-}$ compared to controls (mean ± s.e.m. values; n=3; ****$p < 0.0001$; paired t test; two-tailed; t=6.694, df=12). **(D-H)** Multi electrode array analysis at DIV 50 (mean ± s.e.m.; n=3 independent differentiations; 24 independent wells per differentiation; one-way ANOVA with Tukey's multiple comparisons test; ****$p < 0.0001$; **$p < 0.01$). **(I)** Representative images of hiPSC-derived iNs from family 003 parental control (*ALDH5A1*$^{+/-}$) and patient (*ALDH5A1*$^{-/-}$) at DIV 55 in co-culture with hiPSC-derived astrocytes labeled with DAPI (cyan), MAP2 (green) and TBR1 (magenta). Scale bar 100µm. Quantification of TBR1-positive neurons over MAP2-positive neurons showed no significant difference between all three genotypes used in this study (mean ± s.e.m. values; n=3; ns=non-

significant). **(J)** Western blot of SSADH expression from family 003 iNs at DIV 30. Quantification of SSADH levels from three separate differentiations normalized to β-actin and displayed as a percentage of parental control (*ALDH5A1*$^{+/-}$) (mean ± s.e.m. values; n=3; **p < 0.01) **(K)** Normalized family 003 iNs neurite length per cell-body cluster area [phase] (mm/mm^2) demonstrates significantly longer neurites in *ALDH5A1*$^{-/-}$ compared to controls (mean ± s.e.m. values; n=3; ****p < 0.0001; paired t test; two-tailed; t=5.795, df=12).

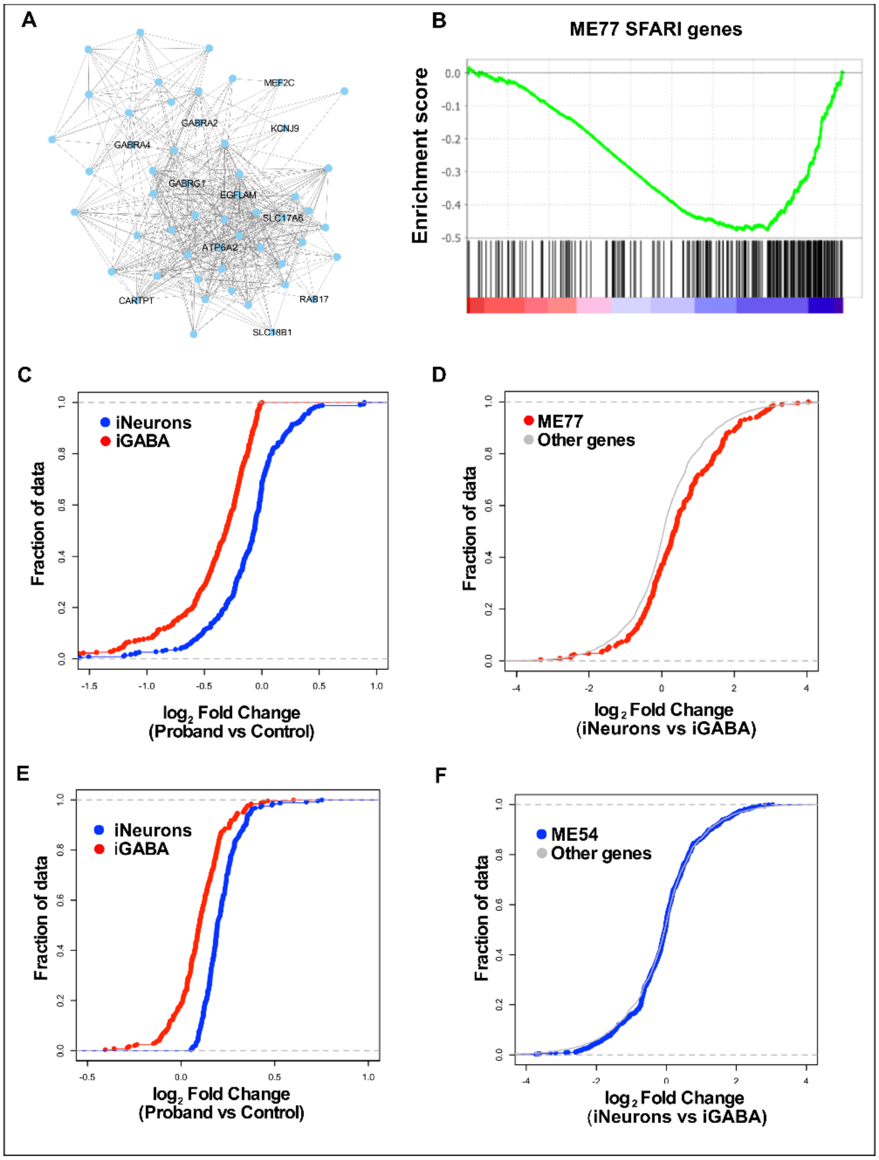

Figure S5. RNA Sequencing in iGABA and iNs

(A) The network plot of the top 1,000 connections within the ME77 module, where each node represents a gene, and the edges represent connections based on Topological Overlap. Genes associated with synaptic function are labeled. **(B)** Gene Set Enrichment Analysis of ASD risk genes obtained from the SFARI database (Category 1) compared to connectivity within ME77 module; n=3 independent differentiations. **(C)** The cumulative distribution function of genes in the ME77 module displays the fold changes comparing proband to control in the iGABA (red) and iNs (blue) neurons. **(D)** The cumulative distribution function of fold changes comparing control iGABA to control iNs for genes in the ME77 module (red) and all other genes (gray). **(E)** The cumulative distribution function of genes in the ME54 module displays the fold changes comparing proband to control in the iGABA (red) and iNs (blue) neurons. **(F)** The cumulative

distribution function of fold changes comparing control iGABA to control iNs for genes in the ME54 module (blue) and all other genes (gray).
